# Optical sensors for operando stress monitoring in lithium-based batteries containing solid-state or liquid electrolytes

**DOI:** 10.1038/s41467-022-28792-w

**Published:** 2022-03-03

**Authors:** Laura Albero Blanquer, Florencia Marchini, Jan Roman Seitz, Nour Daher, Fanny Bétermier, Jiaqiang Huang, Charlotte Gervillié, Jean-Marie Tarascon

**Affiliations:** 1 Collège de France, Chimie du Solide et de l’Energie—UMR 8260 CNRS, 11 Place Marcelin Berthelot, 75005 Paris, France; 2 Réseau sur le Stockage Electrochimique de l’Energie (RS2E)—FR CNRS 3459, 80039 Amiens Cedex, France; 3 Sorbonne Université—UPMC Paris 06, 4 Place Jussieu, 75005 Paris, France; 4 Université Paris-Saclay, Univ Evry, CNRS, LAMBE UMR 8587, 91025 Evry, France

**Keywords:** Energy, Batteries, Characterization and analytical techniques, Imaging and sensing, Electrochemistry

## Abstract

The study of chemo-mechanical stress taking place in the electrodes of a battery during cycling is of paramount importance to extend the lifetime of the device. This aspect is particularly relevant for all-solid-state batteries where the stress can be transmitted across the device due to the stiff nature of the solid electrolyte. However, stress monitoring generally relies on sensors located outside of the battery, therefore providing information only at device level and failing to detect local changes. Here, we report a method to investigate the chemo-mechanical stress occurring at both positive and negative electrodes and at the electrode/electrolyte interface during battery operation. To such effect, optical fiber Bragg grating sensors were embedded inside coin and Swagelok cells containing either liquid or solid-state electrolyte. The optical signal was monitored during battery cycling, further translated into stress and correlated with the voltage profile. This work proposes an *operando* technique for stress monitoring with potential use in cell diagnosis and battery design.

## Abstract (web summary)

Chemo-mechanical stress within Li-based batteries detrimentally affects the performance and lifetime of these devices. Here, the authors propose an operando technique using optical fibers embedded in electrodes for internal stress monitoring of cells containing either solid or liquid electrolytes.

## Introduction

Batteries play a key role in the ongoing energy transition from fossil fuels to renewable energies^1,2^. In particular, rechargeable lithium-ion batteries (LIBs) are currently the dominant technology in strategic industries dealing with consumer electronics, power grids, aerospace, and electrical mobility^3^. Such supremacy comes from their excellent performance meeting most of the energetic demands associated to various applications. However, this technology still needs improvement in terms of its energy density, power rate, lifespan, safety, and environmental footprint. This explains the ongoing efforts focused on (i) the development of new electrodes of higher capacity for electrochemical storage^4^, (ii) new material morphologies and electrode structures for higher power rate^5^, (iii) new chemistries for lowering the sustainability burden^2,6^, and (iv) new cell architectures to enhance performance while increasing safety as it is the case of solid-state batteries^7^ that arose the enthusiasm of our community.

The success of these approaches will depend on various and intertwined parameters: electronic and ionic transport processes, phase transformations, nature and dynamics of the interfaces, and their mechanical integrity. While there is an increasing awareness of the close relationship between chemo-mechanical effects and battery performance, such interplay remains poorly understood^8,9^. Such a lack of understanding is becoming even more critical with the actual development of all-solid-state batteries (ASSB’s) that consist of densified layered stacks exhibiting complex chemo-mechanics at both electrodes and interfaces that cannot be any longer buffered as by liquid electrolytes. Thus, even the small volume changes of the active materials in ASSB’s are transferred across the interfaces and lead to local contact loss or cracking, as shown by synchrotron X-ray tomography and dilatometry studies^10^.

Li-driven volume changes of electrodes in Li-ion batteries with liquid electrolyte have been mainly followed via in situ X-ray diffraction (XRD), *operando* electron microscopy, or dilatometry studies^11,12^. Worth also recalling that the optical bending cantilever technique for determining stresses associated with phase transitions in insertion electrode materials was reported, but mainly applicable to thin-film electrodes on silicon substrate^13,14^. On the other hand, scientists have managed to understand and quantify Li-driven volume and stress changes in ASSBs by successfully placing force sensors in the axial direction of the battery providing new insights on the chemo-mechanical aspects at the cell level^10,15,16^. However, this approach presents two main limitations: 1- only the axial component of the stress can be monitored and 2- only information at the device level is provided, as the sensor is placed outside the cell and thus no decoupling of local phenomena taking place at different electrodes is possible. Hence, the need to develop local and non-invasive *operando* techniques that, combined with specific cell designs, will enable to probe of the chemo-mechanical evolution of battery materials and interfaces under real cycling conditions^9,11^.

On the other hand, battery electrodes that are either an alloyed metal or an intimate mixture of active material, binder, carbon, and eventually solid electrolyte can be conceived as a solid composite matrix. In turn, measuring stresses in composite materials is an old problem that has been worked out for decades by embedding optical Fiber Bragg grating (FBG) sensors within the solid matrix^17^ and correlating the optical signal with temperature (*T*), hydraulic pressure (*P*), and strain (*ε*) changes taking place in the vicinity of the sensor. Such perturbations cause either the shifting of the reflected wavelength and/or the splitting of the signal due to induced birefringence^18^. This approach is routinely applied to a wide variety of load-bearing structures such as bridges and train railways as well as in other composite structures such as containers or reservoirs^19,20^. Such a wide use of FBGs is rooted in the numerous advantages that optical fibers offer like their reduced size (diameter ~150 μm) that makes them non-invasive, their chemical stability in various environments, and their immunity to electromagnetic interferences due to its electrically insulating nature^21^.

Owing to such benefits, FBGs have recently been integrated inside 18,650 and pouch cells for direct internal monitoring of *T*, *ε*, and *P*, and their correlation with the battery’s state of health (SoH) and state of charge (SoC)^22^. More recently, we demonstrated the feasibility of using three well-positioned FBG sensors in 18,650 batteries for performing *operando* optical calorimetry to decode chemical and thermal events under real working conditions^23^. Alternatively, FBGs mounted externally onto the surface of pouch cells were used to monitor strain changes in graphite anodes associated with the various stages of the Li insertion process^24^. Pushing further this approach, Bae et al. implanted FBGs within Li-ion liquid pouch cells to follow the stress evolution in graphite electrodes during Li insertion^25^. Although accessing such electrode breathing observables is essential for enhancing Li-ion battery lifetime, this pioneering work was no longer pursued. This aspect is even more crucial for the upcoming generation of ASSBs that require external pressure for operation and whose performances highly depend on the complex chemo-mechanics and stress variations at the electrodes and interfaces upon cycling.

Inspired by the aforementioned research articles and the widespread industrial use of FBGs in large composite structures for monitoring their mechanical integrity, we decided to further exploit their use in the battery field including Li-based cells containing solid-state or liquid electrolytes. In this work, we report the use FBGs for the internal *operando* monitoring of Li-driven stress changes in InLi_x_ and Li_x_Si electrodes containing either liquid or solid-state electrolytes. Moreover, we show the implementation of FBG sensors at various positions in different all-solid-state cell configurations (InLi_*x*_ | Li_3_PS_4_ | Li_4_Ti_5_O_12_ or InLi_*x*_ | Li_3_PS_4_ | InLi_*x*_) that enables to assess electrodes and interfaces stresses via data analysis relying on both empiric and theoretical models. Additionally, we demonstrate the benefit of this *operando* characterization technique for ASSBs for its local sensitivity, providing insights directly at the material level, which cannot be acquired by external force sensors.

## Results

### Internal stress evolution in cells with liquid electrolyte

**Fig. 1:**
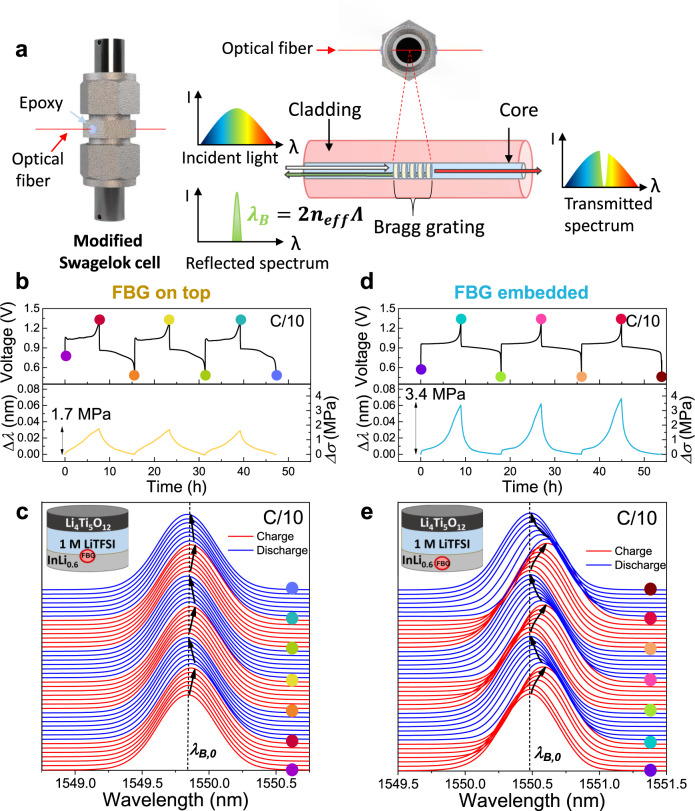
Experimental setup, FBG working principle, and Li-driven stress monitoring in InLi_*x*_ || LTO cells with liquid electrolyte. **a** Scheme of the integration of an FBG into an in-house modified Swagelok cell together with the working principle of an FBG optical sensor. **b** Time-resolved voltage (top) and Δ*λ* and Δ*σ* (bottom) evolution from the FBG sensor of an InLi_0.6_ | 1 M LiTFSI in DOL:DME | LTO cell with liquid electrolyte with the FBG placed at the anode/electrolyte interface. **c** 2D stack-view of the reflected spectra given by the FBG sensor located at the anode/electrolyte interface for the cycles shown in (**b**). **d**, **e** Analogous plots to (**b**, **c**), for a cell with the FBG sensor embedded within the InLi_*x*_ electrode.

In order to track Li-driven stresses in electrode materials, an FBG sensor inscribed in a single-mode optical fiber was first injected through an in-house modified Swagelok cell via two diametrically opposed holes (*Φ* = 800 μm) pierced in its body. Once the battery was assembled, the cell was hermetically sealed on both sides via epoxy glue to fix the fiber at both ends, making the system airtight (Fig. 1a, left). Such modification was shown not to affect the cell electrochemistry (Supplementary Fig. 1). To carry out the measurement, the FBG sensor was either embedded or placed on top of the selected electrode.

When light travels through the optical fiber, the FBG sensor acts as a reflector for a specific wavelength, namely the Bragg wavelength (λ_*B*_) which is defined as *λ*_*B*_ = 2*n*_eff_*Λ*, where *n*_eff_ is the effective refractive index and *Λ* is the Bragg grating period (for short-period grating, normally ~500 nm) (Fig. 1a, right). Any temperature (*T*), hydraulic pressure (*P*), or strain (*ε*) change happening in the surroundings of the FBG sensor will modify either *n*_eff_ and/or *Λ* which will be translated into a variation in the reflected wavelength visualized as a peak shift (Δ*λ*_*B*_). The richness of such a detection becomes in turn problematic when aiming to track only one single physical perturbation. In this regard, successful decoupling of *ε* and *T* variations was achieved via several strategies enlisting either the wise pairing of two FBG sensors or the use of a hybrid FBG/Fabry–Perot cavity sensor^26,27^. On the other hand, commercial FBG sensors are poorly sensitive to hydraulic pressure variations (originated by the gas evolved during cell cycling^23^) due to its non-hollow shape. In order to correlate the optical signal solely to *ε* changes while neglecting contributions coming from *T* and hydraulic *P* variations, we set up the following working conditions. Firstly, the battery testing is done in a temperature-controlled climatic chamber to get rid of any fluctuation in the ambient temperature and negligible temperature changes from the cell during cycling are ensured as demonstrated by a second FBG sensor placed on the surface of the Swagelok cell (see Supplementary Fig. 2 for details). Secondly, we use a low loading (i.e., <8 mg cm^−^^2^) of active material in the positive electrode to minimize the intrinsic heat release and gas production to ensure limited hydraulic pressure. Lastly, to maximize stress changes we decided to work with model materials (In and Si) having a large molar volume difference when alloyed with Li (Δ*Ṽ*_In→InLi_ = +53 %^28^; Δ$\tilde{V}_{{\mathrm Si} \to {\mathrm {Li}}_{15} {\mathrm {Si}_{4}}}$ = +280 %^12,29,30^). Thus, when the FBG is strained under these conditions, the shift of *λ*_*B*_ can be rewritten as^31,32^

1 $$\frac{{\varDelta \lambda }_{B}}{{\lambda }_{B,0}}=\left(1-{\rho }_{e}\right)\varepsilon =\left(1-\frac{{n}_{{{{{\rm{eff}}}}}}^{2}\left[{p}_{12}-\nu \left({p}_{11}+{p}_{12}\right)\right]}{2}\right)\varepsilon$$

where *λ*_*B*,0_ is the Bragg wavelength at the initial time, *ρ*_*e*_ is the effective photo-elastic coefficient, ${p}_{11}$ and ${p}_{12}$ are the strain-optical coefficients of the fiber, and $\nu$ is the Poisson’s ratio. All of these coefficients are well known for silica fibers, with the following material property values: *n*_eff_ = 1.45, $\nu$ = 0.17, ${p}_{11}$ = 0.113, and ${p}_{12}$ = 0.252^18,33^. In turn, measured longitudinal strains can be directly converted into stress by using Hooke’s law: $\sigma =\varepsilon {E}$, where *E* is the Young’s modulus of the silica fiber equal to 69.9 GPa^18,25^. This mathematical treatment will be repeatedly used throughout this work (unless otherwise specified) to convert *Δλ*_*B*_ = (*λ*_*B*_ − *λ*_*B,0*_) into longitudinal strain (*ε*) first, and then translate it into stress (*σ*) expressed in MPa.

### *Operando* stress monitoring in InLi_*x*_ electrodes with liquid electrolyte

In Li-based batteries, strains are the result of several phenomena creating stress within the cell. Relevant stress factors include volume changes of the electrode materials upon cycling due to lithium insertion/extraction, the formation of the solid electrolyte interphase (SEI), and phase transitions of different electrode materials^34^. Bearing this in mind, the In/InLi biphasic system was selected in this work as the model negative electrode material to track electrode stress evolution upon battery cycling, due to the volume change (~53%)^28^ experienced upon alloying (expansion) and dealloying (contraction). More specifically we used biphasic mixtures (1 − *x*)In + *x*InLi; (0< *x* <1) that we denoted hereafter as “InLi_*x*_” for the sake of simplicity. Turning to the positive electrode, the zero-strain material Li_4_Ti_5_O_12_ (LTO, volume changes ~0.2%)^35,36^ was chosen. Thus, InLi_*x*_ || LTO batteries with non-aqueous liquid electrolytes were first assembled in modified Swagelok cells.

Figure 1b shows the galvanostatic charge/discharge voltage profile on an InLi_0.6_ | 1 M LiTFSI in DOL:DME | LTO cell together with the *operando* wavelength shift (Δ*λ*) of the optical signal for an FBG placed at the interphase between the InLi_*x*_ electrode and the glass fiber separator. Thus, the FBG sensor (5 mm length) inscribed within the optical fiber rests on top of the electrode (scheme Fig. 1a, top view) so that under such a fiber positioning any change in the volume of the electrode will strain the FBG and thus trigger a shift in the Bragg peak (*λ*_*B*_). The reflected optical spectra taken in chronological order (from bottom to top) upon battery cycling at C/10 (17.5 mA g^−1^) are reported in Fig. 1c, with the initial *λ*_*B*_ denoted as *λ*_*B*,0_. For all the recorded spectra, a single Bragg wavelength peak is seen, whose maximum (*λ*_*B*_) progressively shifts to the right towards higher values upon charge (red curves) and shifts back to the left upon discharge (blue curves). When looking at successive cycles, a repeated shift back and forth from right to left is also observed, thus revealing the high mechanical reversibility of the system. (Fig. 1b, bottom and Fig. 1c, indicated by arrows).

As previously mentioned, the change in the optical signal is driven by strain-induced on the FBG that is caused by the stress generated in the InLi_*x*_ electrode. Therefore, the shift in the optical signal (Δ*λ*_*B*_) can be translated into electrochemically driven stress changes (Δ*σ*) by using the mathematical model described above (Eq. 1/Fig. 1b, bottom). Δ*σ* well correlates with the electrochemical processes upon subsequent cycling, as it increases during the Li^+^ uptake (volume expansion) and decreases during the Li^+^ release (volume contraction). Interestingly, the Δ*σ* amplitude remains almost constant in the three consecutive cycles shown, which indicates highly reversible processes, evidenced by the good capacity retention. When moving to a slower cycling rate (C/30, 5.83 mA g^−1^), an increase in the total Δ*σ* (from 1.7 MPa to >2 MPa) was observed for each hemicycle, consistent with the higher degree of lithiation obtained at a slower cycling regime (Supplementary Fig. 3)^10^.

For the sake of comparison, we later interrogated the effect of placing the FBG sensor embedded in the core of the InLi_*x*_ electrode rather than on top of its surface. Results were alike in trend. They exhibit a non-linear increase of Δ*σ* upon lithiation reaching to a maximum value (Δ*σ*_max_ = 3.4 MPa) at the end of the charge, and a reverse decrease of Δ*σ* during the subsequent discharge (i.e., delithiation of the InLi_*x*_-based electrode) (Fig. 1d, e). The departure from linear variation is most likely rooted in diffusional limitations of Li^+^ within the electrode that leads to stress propagation delays at the sensor level depending on its positioning. Additionally, the measured Δ*σ*_max_ when the FBG was placed within the InLi_*x*_ core was almost twice the one recorded when the FBG was located at the electrode/electrolyte interface, following the same trend as Bae et al. reported previously^25^. Nevertheless, in such work, the FBG embedded within the electrode exhibited both peak shifting and splitting of the spectra, ascribed by the authors to the accumulation of both transversal and axial stresses. Such change in the spectra shape was not observed in our case (Fig. 1e) even though we chose In-Li alloy rather than a graphite anode to magnify the volume changes at the electrode material. Such a difference does not come as a total surprise as Li-driven anisotropy stresses are more expected for 2D (graphite) rather than 3D (InLi_*x*_) host structures.

### *Operando* stress monitoring in Li_*x*_Si electrodes with liquid electrolyte

Encouraged by the above results, we next decided to expand our sensing approach to the study of Si composite electrodes, which exhibit a 280% volume increase when fully lithiated^12,29,30^. However, Si anodes present an additional complication compared to InLi_*x*_ which is the low loading required for their proper functioning (in our case, the electrode loadings were <1.5 mg cm^−2^ due to electrochemistry purposes), leading to thicknesses ~40 μm, which are far below from the diameter of our fiber (150 μm). Consequently, measurements were solely conducted with the FBG sensor placed on top of the Si electrode composite using in-house modified Swagelok cells as described before. Bearing in mind that the Li-driven insertion process into Si depends on its particle size we have conducted our study using either nano- (40 nm) or micro- (1–5 μm) sized Si powders.

**Fig. 2:**
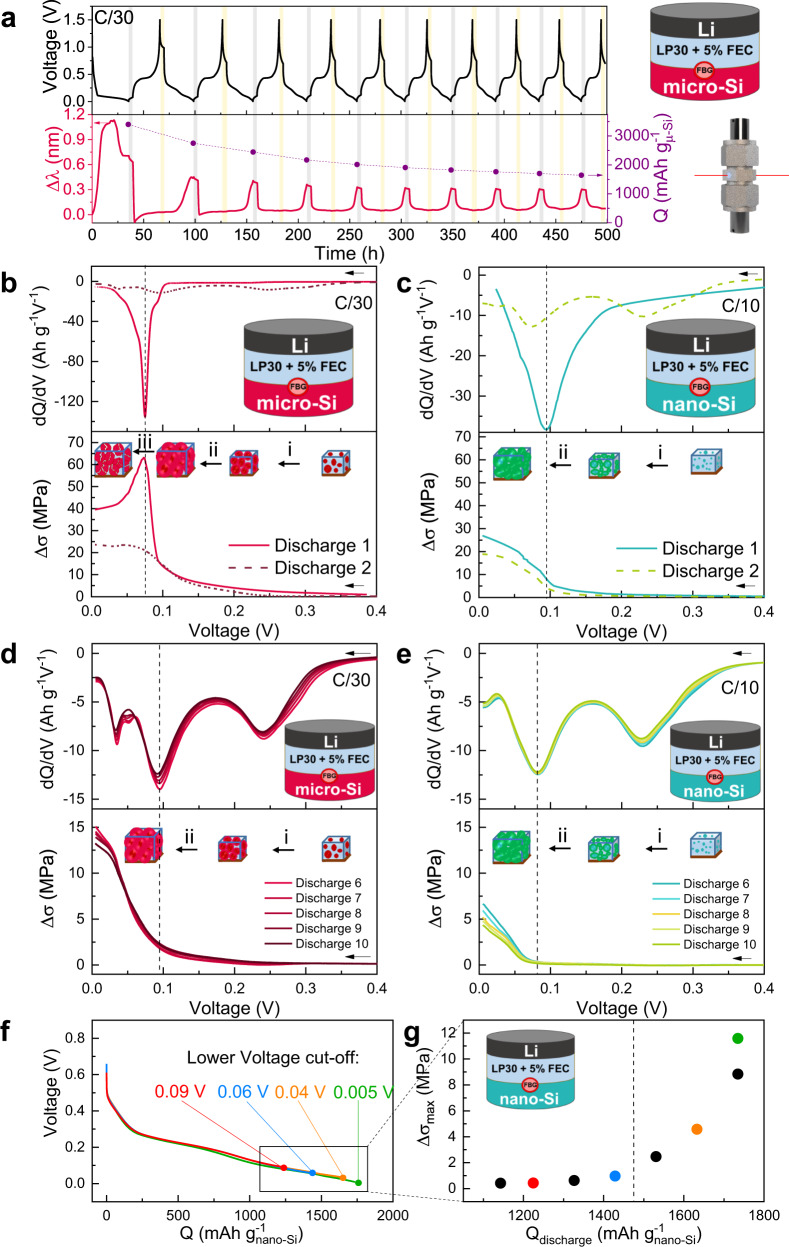
Li-driven stress monitoring in Li || Si cells with liquid electrolyte. **a** Time-resolved voltage profile (top) and *Δλ* (bottom, left) evolution from the FBG sensor of a Li | (LP30+FEC)| Si cell with liquid electrolyte with the FBG placed at the interface between the Si-based electrode and the electrolyte contained in the porous separator. The discharge capacity (bottom, right) is also presented at the end of each lithiation. After each discharge and charge, 6 h of OCV were defined, shadowed in gray and yellow, respectively. **b**, **c** Comparison of the first and second cycle for micro-Si and nano-Si electrodes, respectively. The d*Q*/d*V* plots together with the Δσ evolution from a FBG sensor located on top of the corresponding silicon electrodes are presented. Schemes of the sequential steps (i) porosity filling, (ii) electrode thickening, and (iii) particles pulverization are shown in the figure. The porosity of the nano-Si electrode and micro-Si electrode was 51% and 75%, respectively. **d**, **e** Comparison of the sixth to the tenth cycle for micro-Si and nano-Si electrodes, respectively. The d*Q*/d*V* plots together with the Δ*σ* evolution is shown. **f** Galvanostatic curves of the 12th to 18th cycles for the nano-Si cells with different cut-off voltages together with the corresponding **g** Δ*σ*_max_ for the different capacities achieved. The cells were cycled in a 25 °C oven at a C-rate of C/30 (120 mA g^−1^) for micro-Si and C/10 (360 mA g^−1^) for nano-Si to better compare the cycling conditions in terms of efficient particle surface current density.

Firstly, we assembled a Li | (LP30+FEC) | Si Swagelok cell containing micro-Si particles according to the process detailed in the experimental section, with the FBG sensor placed on top of the Si-based electrode. Figure 2a shows ten cycles of the voltage *vs*. time profile of this cell cycled at C/30 (120 mA g^−1^) together with the variation of the optical signal (Δ*λ*_*B*_) as a function of cycling. Both the cell capacity and the amplitude of the optical signal (Δ*λ*_*B*_) decreased proportionally upon cycling, implying their interlinkage, with the largest decrease being observed between the first and second cycles. A drastic stress evolution difference can equally be visualized between the first and second discharges by plotting the evolution of Δ*σ* calculated from Δ*λ* (Fig. 2b) that could be reminiscent of SEI growth. Whereas the first discharge exhibited a peak at Δ*σ*_max_ = 62 MPa followed by a decay, an almost monotonous increase up to Δ*σ*_max_ = 23 MPa without passing through a maximum was observed for the second discharge.

To account for such differences, let us recall that the lithiation of micro-Si is known to proceed according to the following sequence: Crystalline-Si → amorphous-Li_*x*_Si (Li–Si neighbors filling) → amorphous-Li_*x*_Si (Li–Li neighbors filling) → Crystalline-Li_15_Si_4_ during the first lithiation together with the formation of the SEI^37,38^. Microscopically, three phenomena were reported to take place during the first lithiation for micro-Si-containing electrodes, while the alloying mechanism takes place. There are 1—the electrode pore filling, 2—the electrode thickening, and 3—the particle pulverization^39–41^. The expected stress response in each case will be different, corresponding to a smooth stress increase, a steep stress ramp, and a stress release, respectively. Hence, our measured stress curve during the first lithiation can be interpreted as the sequence of the three aforementioned phenomena (Fig. 2b) with the stress decay after Δ*σ*_max_ = 62 MPa attributed to the pulverization of the silicon microparticles. In the same line, the irreversible pulverization of the microparticles could also explain the monotonous stress increase and the smaller Δ*σ*_max_ registered from the second cycle and on: once the fracturing has occurred, the stress variation amplitude on the subsequent cycles becomes significantly smaller (Δ*σ*_max_ = 23 MPa).

Note that during the first lithiation, the aforementioned reaction scheme provides a single peak in the derivative curve^42^ (d*Q*/d*V*) (Fig. 2b) at the same potential as the one in Δ*σ*_max_ (~0.07 V) regardless of the C-rate (Supplementary Fig. 4). However, when moving toward the second lithiation, the large peak at 0.07 V does no longer mask the other reaction scheme steps, which now pop up as three less intense peaks in the derivative curves (see Supplementary Figure 5), with the one appearing at 0.04 V corresponding to the well-documented crystallization of the Li_15_Si_4_ phase^38^. These results are in accordance with recent acoustic data^43^, which report an intensive acoustic activity at the same d*Q*/d*V* position during the first discharge that strongly decreases upon subsequent cycles. However, we must realize that such a simplified description can be perturbed by the dynamic nature of SEI growth.

In order to support the chemo-mechanical interpretation of the measurements, we assembled Li | (LP30 + FEC) | Si cells using a composite electrode containing nano-Si particles, that are known not to crack nor to trigger the Li_15_Si_4_ crystallization during discharge^41,44^. These cells were cycled at C/10 (360 mA g^−1^) while recording the optical signal. As before, upon cycling the amplitude shift of the optical signal (Δ*λ*_*B*_) mirrors the retention in the capacity decay (Supplementary Fig. 6). However, the cell performance exhibits better capacity retention for nano-Si than those based on micro-Si (Supplementary Fig. 7) confirming the well-established literature trend^45^. Figure 2c shows the d*Q*/d*V* and Δ*σ* evolutions for the first and second lithiation for nano-Si. Note that the minimum in the d*Q*/d*V* plot nearly corresponds to the position at which Δ*σ* starts to drastically increase prior to reaching a maximum value of ~26 MPa at the end of the first lithiation without passing through a maximum, as expected in the absence of cracking/pulverization. Overall, these observations reinforce our claim that the measured stress with the FBG sensor is nested in the volume changes associated with the silicon electrode.

Further analyzing our collected data we next plot for sake of comparison the d*Q*/d*V* and Δ*σ* profiles for cycles six to ten for cells based on either micro-Si (Fig. 2d) or nano-Si (Fig. 2e). First to notice is the full superposition of the curves indicating the good functioning of the electrodes. Interestingly, a peak at 0.04 V was evidenced in the micro-Si d*Q*/d*V* plot while being absent for nano-Si as expected from literature reports and attributed to the crystallization of the Li_15_Si_4_ phase^38,44^. However, in contrast to the expected continuous volume variation upon lithium alloying reaction, neither nano-Si nor micro-Si exhibits a monotonous Δ*σ* increase, but a rather constant response until the last stage of the lithiation, where a drastic increase is observed. To better understand this stress evolution, we conducted several galvanostatic cycles of the nano-Si by progressively reducing the discharge cut-off voltage and by the same varying the amount of Li inserted in the Li_*x*_Si electrode (Fig. 2f). The corresponding measured Δ*σ*_max_ (Fig. 2g) are nearly constant for discharge capacities below ~1400 mAh g^−1^ (*x* ~1.5), while rapidly increasing afterward with decreasing the lower cut-off voltage.

A clue as to the origin of such a capacity threshold beyond which drastic stress is triggered can be found in early work by Bridel et al.^39^. By studying, the Li-driven expansion monitoring of CMC-made Si composites by in situ SEM, the authors revealed that the porosity is acting as a buffer against the Si particles expansion up to *x* = 1.7–2 Li/Si. Beyond that, this buffering effect stops, and the electrode thickness (i.e., volume) starts to rapidly grow, hence leading to a two-step increase in the silicon-based electrode thickness alike the two stress domains we observed using FBGs resting on top of the Si electrode. Such results clearly show the importance of the electrode porosity in ruling the critical capacity value for triggering drastic expansions of the Si electrode, although it must be realized that this value varies due to the SEI partially blocking the porosity. In the same line of thought, we can also explain the increase in Δσ starting at a higher voltage value for micro particles than for nanoparticles (Fig. 2d, e), owing to their copious cracking that rapidly fills out the porosity, in agreement with recent stress measurements on silicon electrode via outside force sensor^16^. Lastly, based on this porosity argument, the nearly linear variation of Δ*λ*_*B*_ upon lithiation in InLi_*x*_ || LTO (see Fig. 1b) is not a surprise since here, the InLi_x_ electrode is free of porosity because it was made directly by using an In and Li foil. Altogether, these results have shown the usefulness of FBG sensors to track the lithiation mechanism in micro-Si and nano-Si electrodes and reveal their differences. Moreover, this also shows the importance of porosity buffer to take up stresses generated from the electrode volume changes.

### *Operando* monitoring of the chemo-mechanical stress in all-solid-state Li-based cells

**Fig. 3:**
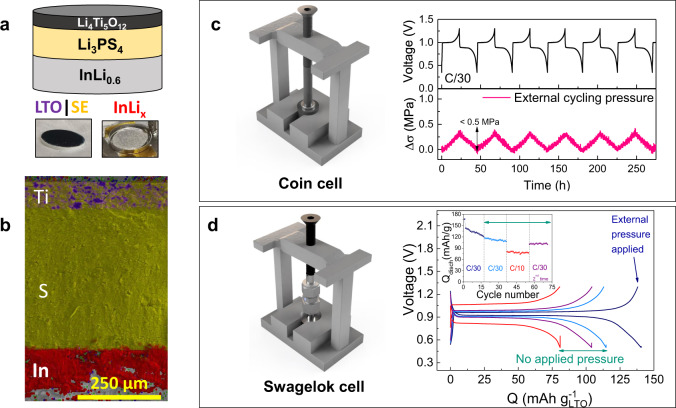
Fiber-free ASSBs tested in the modified cell designs. **a** Scheme of the InLi_0.6_ | Li_3_PS_4_ | Li_4_Ti_5_O_12_ cell and photo of the different battery components. **b** EDX mapping of the cross-section view of the aforementioned ASSB. The selected elements to track the different battery components are Ti (purple) for the cathode, S (yellow) for the solid electrolyte, and In (red) for the anode. The remaining bright area on the bottom corresponds to metallic Li not alloyed. The measured thickness for each layer is 84.98, 429.0, and 147.0 μm, respectively. An EDX mapping of the ASSB with the implemented optical fiber can be seen in Supplementary Fig. 8. **c** Scheme of the coin cell placed under a frame with an external force sensor. The galvanostatic cycling of the ASSB (top) is presented together with the external cycling pressure evolution, monitored with the external force sensor for the ASSB cycled at C/30 (5.83 mA g^−1^) and at 25 °C. **d** Scheme of the Swagelok cell placed under a frame with an external force sensor. Fifth galvanostatic charge/discharge cycle and discharge capacity *vs.* cycle number (inset) for the ASSB with the following C-rate protocol: the cell was cycled first at C/30 under external pressure applied by the force sensor and then, the Swagelok screws were totally tightened so the ASSB was cycled without additional external pressure at C/30, C/10 (17.5 mA g^−1^), and C/30 again up to ~70 cycles in total showing good capacity retention.

To substantiate the benefits of our sensing method towards monitoring stresses in electrode materials we extended it to all-solid-state battery architectures. For proof-of-concept in ASSB, we selected the InLi_0.6_ || LTO cell chemistry, with the liquid electrolyte being replaced by the solid electrolyte Li_3_PS_4_ (LPS) (Fig. 3a). Figure 3b exhibits the SEM-EDX mapping of the cross-section view of an assembled InLi_x_ | LPS | LTO full cell (an EDX mapping of the ASSB with the implemented optical fiber is shown in Supplementary Fig. 8). The selection of a zero-strain insertion LTO positive electrode was not fortuitous but done to strictly monitor the Li-driven stress changes in the InLi_0.6_ electrode under study. For the same reason, LTO was used early on by Janek et al. to facilitate the external tracking of stress changes of NMC electrodes in solid-state batteries by means of an external force sensor^46^. Here we deviate from this approach by monitoring Li-driven internal stresses within an all-solid-state cell by means of FBG sensors placed within the electrode stack. To realize such monitoring, ASSB’s with an integrated fiber were assembled either in modified Swagelok cells as previously described or in modified coin-cells. In both cases, two diametrically opposed holes were made in the respective cell body for the fiber to pass through (see details in Experimental section). Prior to implementing the FBG sensor, we check the suitability of our modified testing cells. Figure 3c shows the galvanostatic cycling for six full cycles together with the external cycling pressure evolution for an ASSB in a modified coin cell, validating the proposed *operando* cell design. On the other hand, Fig. 3d displays the fifth galvanostatic charge/discharge cycle for a modified Swagelok cell cycled at different C-rates. First, the Swagelok cell was cycled at C/30 (5.83 mA g^−1^) under a fixed external applied pressure of 2 MPa. After 20 cycles the Swagelok’s screws were totally tightened and the cell was taken off the frame and being cycled for 20 cycles at C/30, C/10 (17.5 mA g^−1^), and C/30 again up to a total of ~70 cycles. The results proved the proper operation of the modified setups. Thus, for each ASSB studied hereafter, either measurements collected with an internal FBG sensor or an external force sensor will be reported for comparison.

### InLi_*x*_ | LPS | LTO all-solid-state cells

**Fig. 4:**
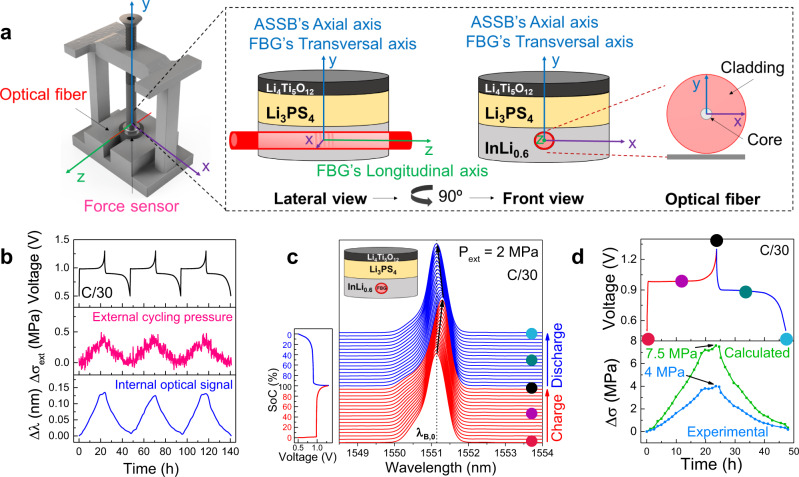
*Operando* Li-driven stress monitoring in InLi_*x*_ | LPS | LTO cell by an FBG embedded in the anode. **a** Scheme of the modified coin cell with the implemented optical fiber and the external force sensor. The corresponding *X*-, *Y-*, and *Z*-axis are detailed in the different views. It is important to note that an axis transverse to the fiber is an axis perpendicular to the main symmetry axis (c∞) and therefore the axis “axial” to the cell is a “transverse” axis to the fiber. To simplify nomenclature, every time we herein mention “longitudinal” or “transversal” will be respected to the fiber and “axial” will only be respected to the cell. **b** Time-resolved voltage (top), external cycling pressure (middle), and an internal optical signal (bottom) for the aforementioned ASSB cycled at C/30 (5.83 mA g^−1^) and 25 °C in an *operando* mode. **c** 2D stack view of the reflected spectra, with the corresponding galvanostatic charge/discharge cycle. The charge and discharge processes are plotted in red and blue, respectively. **d** Comparison between *operando* stress evolution obtained: 1—internally by the FBG sensor and using the mathematical model (green curve) and 2—internally by the FBG sensor and the sensitivity coefficient obtained with the experimental calibration of the sensor (blue curve). The respective galvanostatic charge/discharge is presented (top). The points at the beginning/middle/end of the charge/discharge are indicated by colored dots, also marked in the corresponding FBG spectra in (**d**). The external cycling pressure was fixed at 2 MPa prior to performing the battery cycling.

An InLi_0.6_ | LPS | LTO all-solid-state cell was assembled with an integrated FBG sensor embedded within the InLi_0.6_ anode (Supplementary Figure 9). The system was encapsulated within a modified coin cell case and the cell was then positioned in a metallic frame with a force sensor located at the base, and hermetically sealed with epoxy glue (Fig. 4a). The externally applied pressure was fixed to 2 MPa and the whole setup was finally placed inside a temperature-controlled climatic chamber (±0.1 °C) at 25 °C to proceed with the testing at a rate of C/30 (5.83 mA g^−1^) between 0.5 V and 1.3 V vs. InLi/Li^+^.

The charge–discharge voltage traces of the ASSB, the Bragg peak shifting (Δ*λ*_max_), and the external cycling pressure are depicted in Fig. 4b solely from the third cycle onward because we could not reach stabilization of the mechanical and optical signals through the first two cycles (see Supplementary Fig. 10). A two-dimensional (2D) stack-view of the reflected spectra together with the corresponding state of charge are shown in Fig. 4c for one full cycle at C/30 (5.83 mA g^−1^). The results are alike those obtained for the analogous InLi_*x*_ || LTO cell with the liquid electrolyte shown in Fig. 1e, with a reversible shift in Δ*λ*_max_ toward higher values during charge and a shift back during discharge. This can also be seen directly in the reflected spectra consisting of a single symmetric peak along the whole cycle (Fig. 4c). These changes in Δ*λ*_max_ can be directly converted into stress variations either using the mathematical model based on Hooke’s Law already introduced or through the calibration of our FBG sensors by recording the *λ*_*max*_ while varying the external cycling pressure applied during battery resting at an open-circuit voltage (Supplementary Fig. 11). Results of such calculations are shown in Fig. 4d. Whereas the mathematical model showed a Δ*σ* reaching ~7 MPa at the end of the charge (Fig. 4d, green curve), the stress calculated from the experimental calibration is slightly lower reaching a maximum value of ~4 MPa (Fig. 4d, blue curve). The observed differences indicate an oversimplification of the mathematical model that does not take into account all the experimental parameters. To alleviate this difficulty, we use the linear strain approximation proposed by Janek et al.^46^, which in our case gives us estimated axial stress of ~6 MPa (the detailed calculation can be found in Supplementary Note 1), that is in the order of our experimental data. However, it should be pointed out that in both cases the stress fell back to nearly zero at the end of the discharge, indicating no stress accumulation and hence mechanical reversibility of the system.

In parallel, we also accessed the ASSB’s axial stress at the device level with an external force sensor located in the frame (see Fig. 4b, middle). In this case, the recorded values were far below the ones obtained with internal sensing, barely reaching 0.5 MPa at the end of the charge (see Supplementary Fig. 12 for comparison). This observation clearly shows the importance of local stress measurements for better accessing the mechanical behavior at the component level. In turn, it also highlights the complexity of the stress partitioning in all-solid-state batteries, an aspect that will certainly have to be taken into account in practical systems that are not fully constrained and suffer from plastic deformations.

**Fig. 5:**
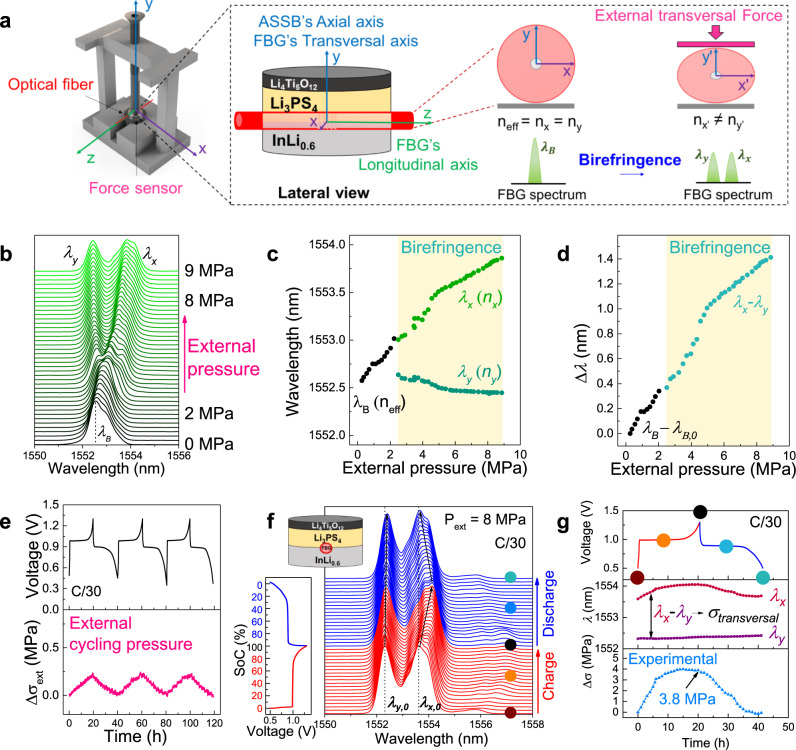
*Operando* Li-driven stress monitoring in InLi_*x*_ | LPS | LTO cell by an FBG placed at the interface between the anode and the solid-state electrolyte. **a** Scheme of the modified coin cell with the implemented optical fiber and the external force sensor. The corresponding *X*-, *Y*-, and *Z*-axis are detailed in the different views. For the sake of simplicity, the “longitudinal” and “transversal” axis is used with respect to the optical fiber, and the “axial” axis is only used with respect to the cell. The scheme of the birefringence phenomena is presented. **b**–**d** Experimental calibration curve of the FBG sensor when the ASSB is in an OCV status. The externally applied pressure is increased externally from 0 to 9 MPa. Detailed values of *λ*_*B*_, *λ*_*x*_, and *λ*_*y*_ *vs.* the externally applied pressure with the force sensor. The birefringence regime is shadowed in light yellow. Two regions are observed: 1—when only one peak is observed in the spectra (*λ*_*B*_, given by *λ*_*B*_ = 2*n*_eff_*Λ*), the calibration is done by *λ*_*B*_ – *λ*_*B*,0_ and 2—the birefringence regime when *λ*_*x*_ and *λ*_*y*_ can be followed. The difference between *λ*_*x*_ and *λ*_*y*_ is used in order to calibrate internal transverse stresses. In our case, we focused on externally applied pressure of 8 MPa to profit from the birefringence phenomenon. Thus, the slope of the linear fitting in the upper birefringence regime (5–9 MPa) is 0.105 nm MPa^−1^. **e** Time-resolved voltage (top), and external cycling pressure (bottom) for the aforementioned ASSB cycled at C/30 (5.83 mA g^−1^) and 25 °C in an *operando* mode. **f** 2D stack view of the *operando* collected spectra by the FBG sensor, with the corresponding galvanostatic charge/discharge cycle. The charge and discharge processes are plotted in red and blue, respectively. **g** Galvanostatic cycle (top), *λ*_*x*_ and *λ*_*y*_ evolution (middle) and o*perando* stress evolution obtained internally by the FBG sensor and with the experimental calibration of the sensor (bottom). The points at the beginning/middle/end of the charge/discharge are indicated by colored dots, also marked in the corresponding FBG spectra in (**f**).

At this stage, a legitimate question regards how the stress evolves at the electrode-solid electrolyte interface, as it reunites two materials of different elasticity, porosity for buffering stresses, and structural morphology. To interrogate this aspect, we decided to assemble a coin-cell type ASSB relying on the same chemistry as above but with the fiber injected between the InLi_*x*_ negative electrode and the LPS electrolyte layer (Fig. 5a and Supplementary Fig. 13, see “Experimental” section for assembling details). We first calibrated our sensor under such configuration by monitoring changes in the FBG optical signal while increasing the externally applied pressure from 0 to 9 MPa. Figure 5b shows a drastic variation in the optical signal with namely a single optical resonance peak (*λ*_B_) at low pressure (0–2.5 MPa) that splits into two peaks whose respective distance continuously increases with increasing the pressure till 9 MPa. This effect could also be artificially induced by simply applying a transversal load to an FBG sensor^47^ placed in between two stainless steel plates (see Supplementary Figure 14). This phenomenon is known as birefringence and it is nested in a physical stimulus-driven elliptical deformation of the optical fiber (see scheme in Fig. 5a) also observed in FBGs embedded in composite materials^17,18,48,49^. Such symmetry break causes the initial effective refractive index to be no longer isotropic but anisotropic leading to two different refractive index components (*n*_*x*_ and *n*_*y*_) with the change in the x-polarization (*n*_*x*_) being much greater than the one for the y-polarization (*n*_*y*_). This explains the splitting of the single resonance peak (*λ*_*B*_, given by *λ*_*B*_ = 2*n*_eff_*Λ*) of an FBG into two peaks (*λ*_*x*_ and *λ*_*y*_) when the sufficiently high transversal load is applied onto the FBG sensor.

The birefringence (*B*) of the light propagating through the optical fiber is given by^50^

2 $$B=\frac{\left|{n}_{{{{{{\rm{||}}}}}}}-{n}_{\perp }\right|}{{n}_{{{{{\rm{eff}}}}},o}}={B}_{0}+\frac{\left|{\triangle n}_{{{{{{\rm{y}}}}}}}-{\triangle n}_{{{{{{\rm{x}}}}}}}\right|}{{n}_{{{{{\rm{eff}}}}},o}}$$

where ${n}_{{||}}$ and ${n}_{\perp }$ are the refractive index in the parallel and perpendicular direction of the externally applied load, *Δn*_*x*_ and *Δn*_*y*_ are the refractive index changes for the x- and y-light polarizations, respectively, due to the external applied load and *n*_eff,0_ is the initial effective refractive index. *B*_0_ is the birefringence induced by the manufacturing of the grating, which for low-birefringence FBG sensors is neglected. From our results, we could extract *λ*_*x*_ and *λ*_*y*_ based on specific criteria (see Supplementary Fig. 15) to do this decoupling when the splitting was either well or not well defined. From the calibration curve performed at OCV, the decoupled *λ*_*x*_ and *λ*_*y*_ resonance peaks are reported as a function of the external cycling pressure, with their difference in wavelength becoming larger with increasing pressure (Fig. 5c, d). Such a variation is not fortuitous but contains information regarding the transversal stresses experienced by the FBG sensor, and so happening in the axial axis of the ASSB. Hence, this birefringence phenomenon provided by FBGs offers a powerful analytical tool to obtain greater insights on the directional (longitudinal vs. transversal) stress taking place at the electrode–solid electrolyte interface in an all-solid-state battery under practical operating conditions. Hereafter, for nomenclature simplification in specifying stress direction, we are using “longitudinal” or “transversal” in reference to the fiber and “axial” for the battery (see schemes in Fig. 4a and Fig. 5a).

We explored the benefits of birefringence within ASSB, by studying the Li-driven stress changes in an InLi_*x*_ | LPS | LTO coin cell cycled at C/30 (5.83 mA g^−1^) under an applied external pressure of 8 MPa (Fig. 5e, f) where the two peaks could be properly identified. Figure 5f shows the 2D stack-view of the reflected spectra given by the FBG sensor and the corresponding time-resolved voltage profile for one cycle. *λ*_*x*_ and *λ*_*y*_ were followed during charge and discharge showing high reversibility following the electrochemical signal (Fig. 5g). From the peak separation (*λ*_*x*_ − *λ*_*y*_) and the slope obtained from the FBG calibration (Fig. 5d), we could calculate the transversal interfacial stress (Δ*σ*) for this specific InLi_*x*_–LPS interface, that increases nonlinearly to peak at 3.8 MPa by the end of the first charge. Upon subsequent discharge, the stress returns almost to zero also in a non-fully linear way, likely due to Li^+^ diffusion limitations in the alloy. As in the previous case, the ASSB’s axial stress at the device level was also monitored with an external force sensor with the recorded values (<0.5 MPa) being underneath the obtained with internal sensing (Fig. 5e).

When looking deeper into the shape of the reflected spectra, the onset of a third peak alongside *λ*_*x*_ (*λ* ~1554 nm) can be visualized. Such an additional shoulder is a common feature pertaining to FBG’s responses. Several studies have been carried out to characterize such a spectral response and it was demonstrated, via combined optical measurements, simulations, and mathematical treatments that it is due to a highly non-homogeneous transverse stress field distribution along the FBG sensor including both transversal and longitudinal contributions^51–53^. Thus the real complexity in properly identifying the origins of the detailed features present in the optical signals. Although pushing optical sensing to such a limit could be useful, it is beyond the scope of this paper.

### InLi_*x*_ | LPS | InLi_*x*_ symmetric all-solid-state cell

**Fig. 6:**
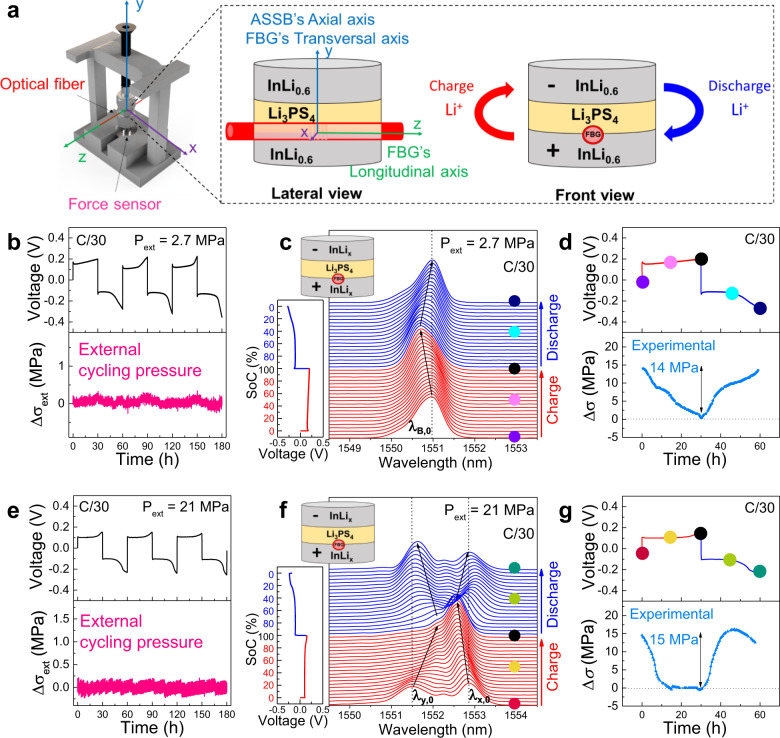
*Operando* Li-driven stress monitoring in a symmetrical InLi_*x*_ | LPS | InLi_*x*_ cell by an FBG located at the interface between the cathode and the solid-state electrolyte. **a** Scheme of the modified Swagelok cell with the implemented optical fiber and the external force sensor. The corresponding *X-*, *Y-*, and *Z-*axis are detailed in the different views. For the sake of simplicity, the “longitudinal” and “transversal” axis is used with respect to the optical fiber, and the “axial” axis is only used with respect to the cell. The direction of the Li^+^ ions during charge/discharge is detailed in the scheme. **b** Time-resolved voltage (top), and external cycling pressure (bottom) for the aforementioned ASSB cycled at C/30 (5.83 mA g^−1^) and 25 °C for three consecutive cycles, at an externally applied pressure of 2.7 MPa, and **e** 21 MPa. **c**, **f** 2D stack view of the collected spectra by the FBG sensor, with the corresponding galvanostatic charge/discharge cycle when the externally applied pressure is 2.7 and 21 MPa, respectively. The charge and discharge processes are plotted in red and blue, respectively. **d** Galvanostatic cycle (top), and o*perando* stress evolution obtained internally by the FBG sensor and with the experimental calibration of the sensor (bottom) when the externally applied pressure is 2.7 MPa, and **g** 21 MPa. The points at the beginning/middle/end of the charge/discharge are indicated by colored dots, also marked in the corresponding FBG spectra in (**c**). Note that due to the location of the FBG sensor in the positive electrode, the relative stress is normalized (Δ*σ* = 0 MPa) at the beginning of the discharge to compare positive stress variations.

Aiming to validate the added value of the internal stress sensing using implanted FBGs over the external sensing using a force sensor, we assembled symmetrical all-solid-state InLi_*x*_ | LPS | InLi_*x*_ Swagelok cells (see scheme in Fig. 6a) and the stress was monitored simultaneously with the two methodologies. In this case, the FBG was placed at the interphase between one InLi_*x*_ electrode and the solid-state electrolyte, and the cell was tested at two different external cycling pressure regimes defined as low (2.7 MPa), and high (21 MPa).

Figure 6b shows respectively the voltage profile of the cell cycle under the pressure of 2.7 MPa together with the variation of the external cycling pressure measured with the force sensor. To no one’s surprise, the force recorded externally showed almost no variation upon cycling. This was expected given the symmetry of the electrochemical system, meaning that the stress increase in one electrode should correspond exactly to the stress release in the other, making the whole device to be in apparent mechanical equilibrium. However, when looking at the optical response (*λ*_*B*_, Fig. 6c) of the FBG sensor during a full charge–discharge cycle at C/30 (5.83 mA g^−1^), there is a reversible shift from right to left matching the charge and discharge stages, as observed for the batteries previously shown (see Fig. 4 and Fig. 5). We later translated Δ*λ*_*B*_ into stress variations (Δ*σ*) with an experimental calibration curve (Supplementary Fig. 16) and noted a repeated stress change with a maximum amplitude of around 14 MPa (Fig. 6d). Overall, this result demonstrates that FBGs integrated into battery electrodes provide insights at the material level thanks to their sensitivity to local stress variations, thus opening a playground in the *operando* monitoring of mechanical properties that goes beyond the average changes of the whole device.

Next, we manually increased the external cycling pressure to 21 MPa while the cell was at OCV, recording the corresponding calibration curve (Supplementary Fig. 17). As before, upon cycling, no changes in the force monitored externally were recorded (Fig. 6e). However, the compression to 21 MPa caused the optical signal to drastically change and eventually induced birefringence, evidenced in the presence of two resonance peaks (*λ*_*x*,0_ and *λ*_*y*,0_) instead of one (Fig. 6f). We monitored the variation of the two peaks as a function of cycling and noted that they were repeatedly merging in one and splitting again upon charging and discharging the cell, thus indicating local anisotropic Li-driven stresses that are reversible upon cycling (Fig. 6f, g, and Supplementary Fig. 18). The transversal stress was later determined from the difference between *λ*_*x*_ and *λ*_*y*_ and the experimental calibration curve, reaching a maximum value of about 15 MPa (Fig. 6g). This finding is consistent with the recent *operando* synchrotron radiation X-ray tomographic microscopy report, which indicates the higher stress contribution is coming from the axial axis of the cell that is equivalent to the fiber transversal axis^54^. Moreover, we noted that the measured stress was nearly independent of the applied stack pressure in agreement with previous work^16^ while it enables to reduce the cell polarization, hence favoring the charge transfer (Fig. 6b vs. Fig. 6e). Although these results show the great potential of FBG sensing in spotting local mechanical stress, a great amount of work remains to be done to precisely ascribe the origin of these stresses in such mechanically complex systems as composite electrodes.

## Discussion

In summary, we have investigated the use of FBG sensors for non-invasive *operando* monitoring of Li-driven stresses in electrodes contained into Swagelok or coin cells comprising a liquid or solid-state electrolyte. For proof-of-concept, we have selected Li-alloying electrodes that are known to undergo large volume changes upon Li uptake or removal. By monitoring the variation of the optical wavelength signal (Δ*λ*_*B*_) during cycling and converting it into Δ*σ* we could access quantitatively to Li-driven local stresses at the electrode level, which has never been achieved so far in ASSB’s with external force sensors. Throughout stress monitoring by FBG sensors, we also succeeded in differentiating the electrode behavior of nano *vs*. micro Si particles towards Li uptake while reminding the importance of porosity in buffering electrode expansion, hence providing clues in determining the proper cycling range for minimizing capacity loss. Moreover, by taking advantage of the birefringence phenomena we demonstrated the feasibility to access the directional anisotropy of the Li-driven stress field when the FBG sensor is placed at the solid InLi_*x*_ | LPS interface, reuniting two materials of different elasticity. Lastly, we showed that external force sensors were totally blind to stress events occurring at the electrode level in symmetric InLi_*x*_ | LPS | InLi_*x*_ cells (showing constancy of Δ*σ* upon cycling) while FBGs placed in the interphase between the InLi_*x*_ electrode and the solid-state electrolyte LPS successfully tracked the electrode’s stress variations during cycling, hence highlighting the benefits offered by internal rather than external stress monitoring in all-solid-state batteries.

Altogether, internal stress diagnostic via FBG sensors has the potential to offer great opportunities within the battery field both at the fundamental level to get insights on chemo-mechanical processes at the interfaces and within electrodes, and on practical aspects oriented to enhance the performance of Si-based electrodes and ASSBs. However, for this to happen several remaining difficulties must be resolved. These range from the design of suitable cell hardware enabling an easier integration and positioning of the FBG sensors within the cell components to theoretical calculations of the Li-driven variation of material mechanical properties (such as Young’s modulus) for a deeper interpretation of the observed stress evolution. Extensions of this work enlist 1—the study of layered compounds, materials of choice by virtue of their 2D structure, for further digging into the science beyond chemo-mechanical aspects and 2—testing the efficacy of self-healing electrodes that are of paramount importance for LIBs. We speculate that our present findings together with future developments could play a key role in properly selecting and pairing suitable electrode materials for facilitating the development of all-solid-state batteries.

## Methods

### Materials and electrode preparation

#### Preparation of the Li_4_Ti_5_O_12_-based electrodes and non-aqueous LiTFSI-based liquid electrolyte solution

Lithium titanate (Li_4_Ti_5_O_12_, LTO) electrodes were prepared to adapt the experimental protocol from Singh et al.^55^ using 87.9% LTO, 4.8% Super P conductive carbon black (Csp, 99%) and 7.2% polyvinylidene fluoride type PVDF-HFP 1800 2801-00 (Kynar) which was dissolved in 6 mL of 1-methyl-2-pyrrolidone (N-methyl-2-pyrrolidone, NMP, 99.5%, Sigma-Aldrich Chemie GmbH) with a resulting concentration of 36 mg L^−1^. The LTO and the carbon black were hand-mixed in a mortar before adding the dissolved PVDF binder under continuous stirring. The slurry was then cast by hand onto the aluminum foil (99.95%) using a doctor blade (thickness of 100 µm). After drying at 80 °C for 1 h the electrodes were punched using an 11 mm punch, resulting in electrodes of 250 ± 5 µm thickness and mass loading of 4.5 ± 0.1 mg cm^−2^. Before passing the electrodes to the glovebox, they were again dried at 100 °C for 12 h in a B-585 vacuum oven (BüchiLabortechnik AG, Germany). 750 µL of 1 M Lithium(I) Bis(trifluoromethanesulfonyl)imide (Li+(CF_3_SO_2_)_2_N−, LiTFSI, obtained from Solvay and used as received) in 1:1 dioxolane (DOL) dimethyl ether (DME) (99.9 %, Solvionic) were used as the liquid electrolyte (H_2_O < 15 ppm), as suggested by Bridel et al. and Santhosha et al.^28,56^.

#### Preparation of the Si-based electrodes, Li metal electrodes, and non-aqueous LiPF_6_-based electrolyte solution

Microparticle-sized silicon powder (micro-Si, 1–5 µm, Alfa Aesar) and nanoparticle-sized silicon powder (nano-Si, 40 nm) were used as active materials. Super P conductive carbon black (Csp, 99%) was purchased from Alfa Aesar, and poly(acrylic acid) (PAA, Mw ≈ 450,000) was obtained from Sigma Aldrich. The silicon anodes were prepared through a slurry casting process. Silicon active material (micro-Si or nano-Si), Csp and PAA, were first hand-milled in the air in a weight ratio of 2:1:1, and milliQ water was then added to the powder mix to achieve a dry mass ratio between 15 and 25%. After 24 h stirring, the homogeneous slurry was casted onto the copper foil (99.9%, thickness of 26 µm) using a doctor blade (Elcometer, 200 μm gap) and then dried for 24 h in a vacuum oven at 80 °C. The resulting micro-Si and nano-Si based electrodes presented an average thickness and mass loading of 1.1 ± 0.1 mg cm^−2^ and 23 ± 1 µm, and 0.5 ± 0.1 mg cm^−2^ and 14 ± 4 µm, respectively. LP30 electrolyte (99.9%, Solvionic, stored in the glovebox and used as received), composed of 1 M Lithium hexafluorophosphate (LiPF_6_) in EC:DMC (1:1 by volume), was used to prepare our LP30+FEC electrolyte (H_2_O < 15 ppm) by adding 5 wt% of 4-fluoro-1,3- dioxolan-2-one (FEC, 98%, Alfa Aesar, used as received). Lithium metal (99.9%, Sigma Aldrich, 0.38 mm thickness) was used as the counter electrode, after being punched with a 5 mm stainless steel punch.

#### Preparation of the indium–lithium alloy (InLi_*x*_) electrodes

Disks were cut from indium foil (99.99%, Sigma Aldrich) with a thickness of 0.127 mm and lithium foil (99.9%, Sigma Aldrich) with a thickness of 0.38 mm in size so that the molar ratio between the two metals was approximately InLi_0.6_. The two metal disks were then placed on top of each other and cold-pressed with a hydraulic press at a pressure of ~1 ton for 1 min to perform the alloy reaction inside an Ar-filled glovebox with H_2_O and O_2_ contents below 0.1 ppm. The formation of the InLi_*x*_ phase was confirmed by XRD measurements (Supplementary Fig. 19).

#### Preparation of Li_4_Ti_5_O_12_-based composite for testing in all-solid-state cell configuration

The cathode composite Li_4_Ti_5_O_12_:Li_3_PS_4_:C (LTO:LPS:C; 30:60:10 wt%) was prepared by hand-mixing the powders with a mortar and a pestle inside an Ar-filled glovebox with H_2_O and O_2_ contents below 0.1 ppm. Lithium phosphorus sulfide (Li_3_PS_4_, LPS) (NEI corporation) was the solid electrolyte used for all the all-solid-state cells.

### Cell assembly and testing

#### FBG sensor implementation in Swagelok cells with Si-based electrodes and liquid electrolyte

Electrodes of 11 mm diameter were punched on the dried films and used to assemble Swagelok-type cells in an Ar-filled glove box with H_2_O and O_2_ contents below 0.1 ppm. First, the electrode of the study was placed on top of a plunger perfectly aligned with the two holes drilled in the Swagelok’s body allowing the optical fiber implementation and the positioning of the 5 mm FBG length on top of the electrode (see Fig. 1a, top view). Then, the subsequent sealing with epoxy glue (Bühler EpoKwick FC) is done by applying the epoxy at each hole of the Swagelok body. Once the glue is perfectly dried, the assembly of the cell is done as in routine Swagelok cells, using two Whatman papers as separator (Whatman GF/D, 650 µm, 12 mm diameter), soaked with 750 µL of the corresponding electrolyte, and Li metal foil as the counter electrode (0.38 mm thickness, 5 mm diameter). All the cells were tested at least twice.

#### FBG sensor implementation in Swagelok cells with InLi_*x*_ electrodes and liquid electrolyte

For the FBG placed on top of the electrode, the prepared InLi_x_ alloy (thickness and diameter of 170 µm and 11 mm, respectively) was placed on top of the plunger and the assembly of the cell was performed exactly as we detailed in the Silicon section. For the FBG embedded in the electrode, first, the lithium foil is placed on top of a plunger perfectly aligned with the two holes drilled in the Swagelok’s body to allow the optical fiber implementation. On top of this configuration, we positioned the indium foil. The three components (lithium/FBG/indium) are cold-pressed together with a hydraulic press to perform the alloy reaction meanwhile the FBG sensor is perfectly embedded. Epoxy glue is then applied to the two drilled holes in the Swagelok’s body (see Fig. 1a). Once the epoxy is dried, the cell is finalized following the routine protocol for Swagelok’s assembly.

#### Fiber Bragg grating sensor implementation in InLi_*x*_ || LTO all-solid-state coin cells

To assemble the cells, 100 mg of Li_3_PS_4_ were first pressed at 1 ton cm^−2^ into a pellet (die set diameter = 13 mm) by a hydraulic press inside an Ar-filled glovebox with H_2_O and O_2_ contents below 0.1 ppm during 30 s. Then, 21.9 mg of LTO cathode composite were added and the pellet was re-pressed at 4 ton cm^−2^ with the hydraulic press for 15 min. On the other hand, the anode was prepared by pressing together 120 mg of indium foil and 4.2 mg lithium foil with a hydraulic press (Manual hydraulic press ATLAS Specac up to 15 ton) at 1 ton for 1 min, giving a final composition of InLi_0.6_. The alloy was formed on top of a stainless steel spacer and placed inside the pre-drilled case of the coin cell (*Φ* = 800 μm). Next, the optical fiber was passed through the holes of the modified coin cell and adjusted making sure the FBG grating is on top of the electrode (see Supplementary Fig. 13). For the FBG embedded in the anode, first, the lithium foil is placed on top of a stainless steel spacer, which is already inside the coin cell case. Then, the optical fiber is passed through the coin cell case holes. Finally, the indium foil is put on top and the formation of the InLi_*x*_ alloy, with the FBG embedded, is done under the hydraulic press. Finally, the LPS/LTO pellet (see the photo in Fig. 3a) was placed on top of the anode alloy. The coin cell was finished by the spacer/spring/cap and sealed with epoxy under the stainless steel frame with the external force sensor (Miniature button load cells up to 5000 N, Applied Measurements Ltd.).

#### FBG sensor implementation in InLi_*x*_ || InLi_*x*_ symmetric all-solid-state Swagelok cells

The body of the Swagelok cell was directly used as the die set to press the solid electrolyte. Therefore, 140 mg of Li_3_PS_4_ were firstly loaded into the body, and cold-pressed at 4 ton cm^−^^2^ for 15 min under a hydraulic press (Manual hydraulic press ATLAS Specac up to 15 ton) inside an Ar-filled glovebox with H_2_O and O_2_ contents below 0.1 ppm. The pellet position was perfectly aligned with the two drilled holes (*Φ* = 800 μm) to alloy the subsequent optical fiber implementation at the interface LPS/InLi_x_ cathode. The holes were sealed with epoxy glue and the cell closed as explained above. The Swagelok cell was positioned under the stainless steel frame including the external force sensor.

For the three cell configurations explained, the respective blanks were assembled with the same procedure omitting the optical fiber implementation.

### Electrochemical tests

The cells were cycled with a BCS-810 or MPG2 potentiostat (Bio-Logic, France) at a constant temperature of 25 °C inside temperature-controlled climatic chambers (Memmert, accuracy ±0.1 °C). The electrochemical performances of the cells were studied by galvanostatic discharge–charge cycling in the voltage range of 0.005–1.5 V vs. Li/Li^+^ for the silicon-based cells and a voltage window of 0.5–1.3 V vs. InLi/Li^+^ for the InLi_*x*_ || LTO cells. The cycling of the symmetrical ASSB was limited by time, with a 30 h step for each charge/discharge.

### Optical measurements

The reflected spectra were collected with the interrogators FBGuard1550 (Safibra, Czech) and LUNA Si255 (Micron Optics, USA). According to the specifications, the wavelength accuracy/resolution of both of them is 1 pm. The FBG sensors (5 mm length, 150 μm diameter) were purchased from SAMYON company (China) and IDIL (France).

### Characterization techniques

#### Scanning electron microscopy (SEM) and energy-dispersive X-ray spectroscopy (EDX)

A high-resolution scanning electron microscope (Oxford Instruments) was used to perform the cross-section micrographs of pristine ASSBs. The pellets were carefully and sharply cut after being previously embedded in conductive epoxy. EDX of the pellets was performed under an acceleration voltage of 20 kV.

#### Laboratory X-ray powder diffraction (XRD)

Laboratory XRD was performed in an airtight cell equipped with a Be window. XRD patterns were recorded in reflection mode in Bragg–Brentano geometry using a Bruker D8 Advance diffractometer equipped with a Cu-Kα X-ray source (λ1 = 1.54056 Å, λ2 = 1.54439 Å) and a LynxEye detector.

#### Electrode porosity

The porosity of the electrodes was estimated by comparing the actual volume of an electrode to its expected volume regarding the true density of each material. This density was measured on a helium pycnometer with a Micromeritics AccuPyc 1330 and Helium Messer gas (≥99.996 vol%). One analysis is composed of 20 helium purges followed up by 5 runs of measurements.

## Supplementary information

Supplementary Information

## Data Availability

The authors declare that the main data supporting the findings of this study are available within the article and its Supplementary Information. Extra data are available on reasonable request from the corresponding author.
