# Supplementary material for: Optical sensors for operando stress monitoring in lithium-based batteries containing solid-state or liquid electrolytes
Source: Nat Commun. 2022 Mar 3;13:1153. doi: 10.1038/s41467-022-28792-w (PMC8894478; doi:10.1038/s41467-022-28792-w)
Supplement: Supplementary file 1 — Supplementary Information [file 41467_2022_28792_MOESM1_ESM.pdf]

Supplementary Information

Optical sensors for operando stress monitoring in lithium-based batteries containing solid-state or liquid electrolytes

Laura Albero Blanquer[1,2,3], Florencia Marchini[1,2], Jan Roman Seitz[1,2], Nour Daher[1,2], Fanny Bétermier[1,2,4], Jiaqiang Huang[1,2], Charlotte Gervillié[1,2], and Jean-Marie Tarascon[1,2,3,*]

[1] Collège de France, Chimie du Solide et de l'Energie - UMR 8260 CNRS, 11 Place Marcelin Berthelot, 75005 Paris, France

[2] Réseau sur le Stockage Electrochimique de l'Energie (RS2E) - FR CNRS 3459, 80039 Amiens Cedex, France

[3] Sorbonne Université – UPMC Paris 06, 4 Place Jussieu, 75005 Paris, France

[4] Université Paris-Saclay, Univ Evry, CNRS, LAMBE UMR 8587, 91025 Evry, France

* Corresponding author

J.-M. Tarascon: jean-marie.tarascon@college-de-france.fr

SUPPLEMENTARY INFORMATION

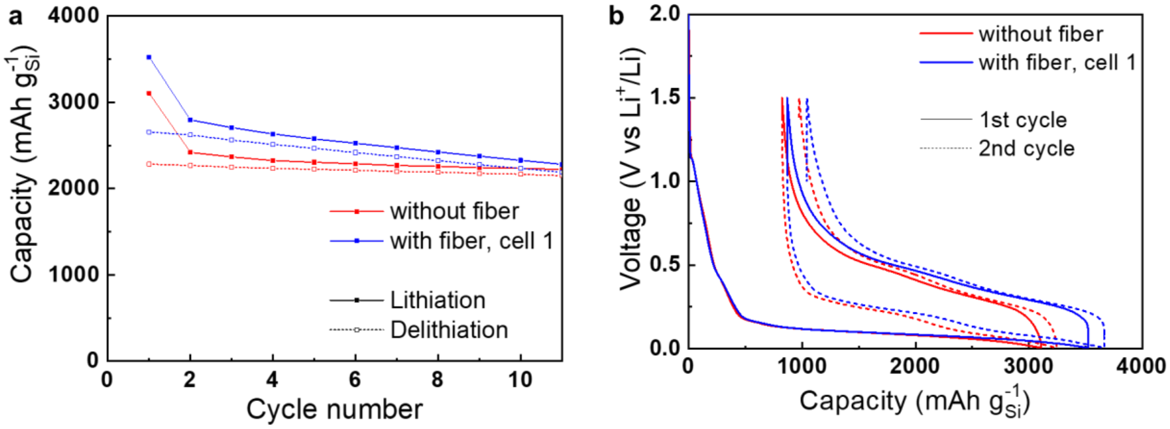

Supplementary Figure 1 | Cycling performance of two-electrode Swagelok cells with liquid electrolyte with and without the optical fiber.

a, **b** Comparison of the battery performance for liquid Li | LP30+FEC | Silicon Swagelok cells with and without the optical fiber. The cells were cycled at C/10 (360 mA g^{-1}) for 10 cycles in a 25 ºC temperature-controlled climatic chamber. The electrochemistry remains unaffected by the presence of the optical fiber.

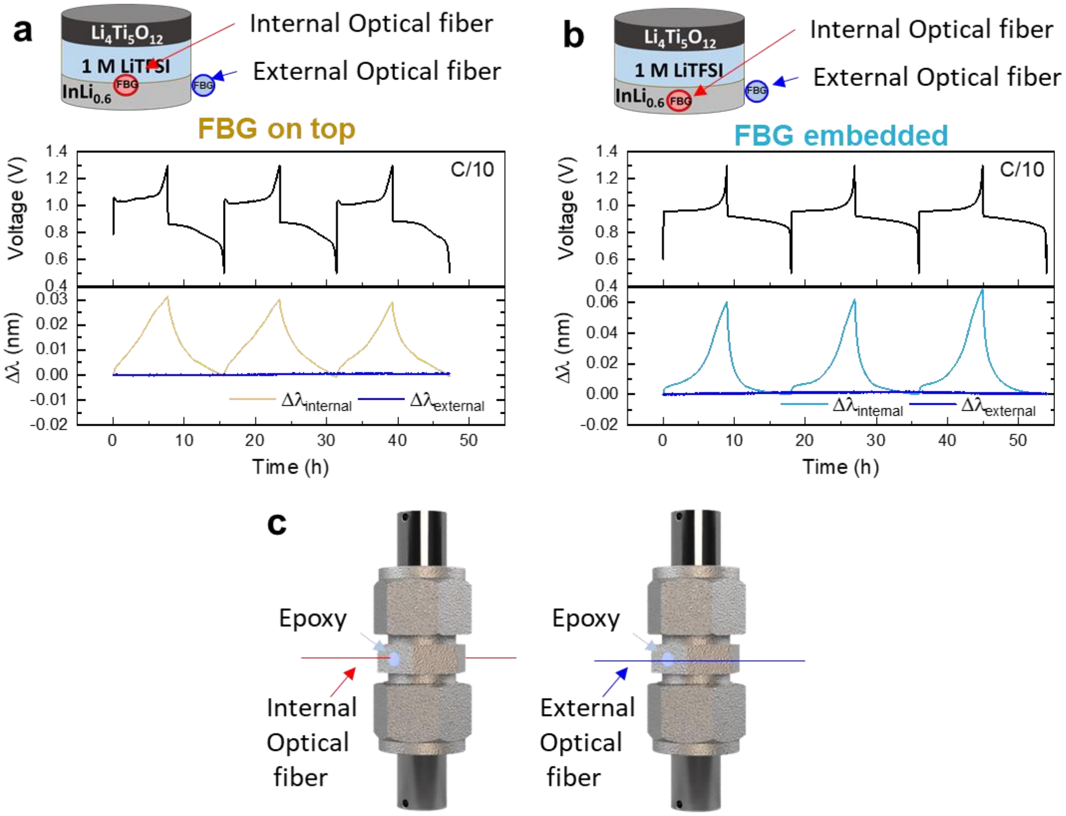

Supplementary Figure 2 | Internal and external FBG *operando* monitoring of the $InLi_x$ || LTO cells with the liquid electrolyte.

a Time-resolved voltage (top) and internal + external optical signal (bottom) for the $InLi_x$ || LTO cells with liquid electrolyte shown in Figure 1, with the internal FBG sensor placed on top of the anode and **b** embedded within the anode. The external optical signal corresponds to a FBG sensor placed on the external surface of the cell to check any thermal heat release variation. The results clearly indicate the negligible variation of the external FBG sensor ($\Delta\lambda_{external}$ < 2 pm) and thus, the insignificant temperature change (< 0.2 ºC), justifying the validity to neglect T. The cells were cycled at C/10 (17.5 mA g^{-1}) and in a temperature-controlled climatic chamber at 25 ºC in an *operando* mode. **c** Scheme of the integration of the FBG sensors in the Swagelok cell. The internal optical fiber and the external optical fiber, the latter one placed aligned to the internal one, but on the surface of the cell.

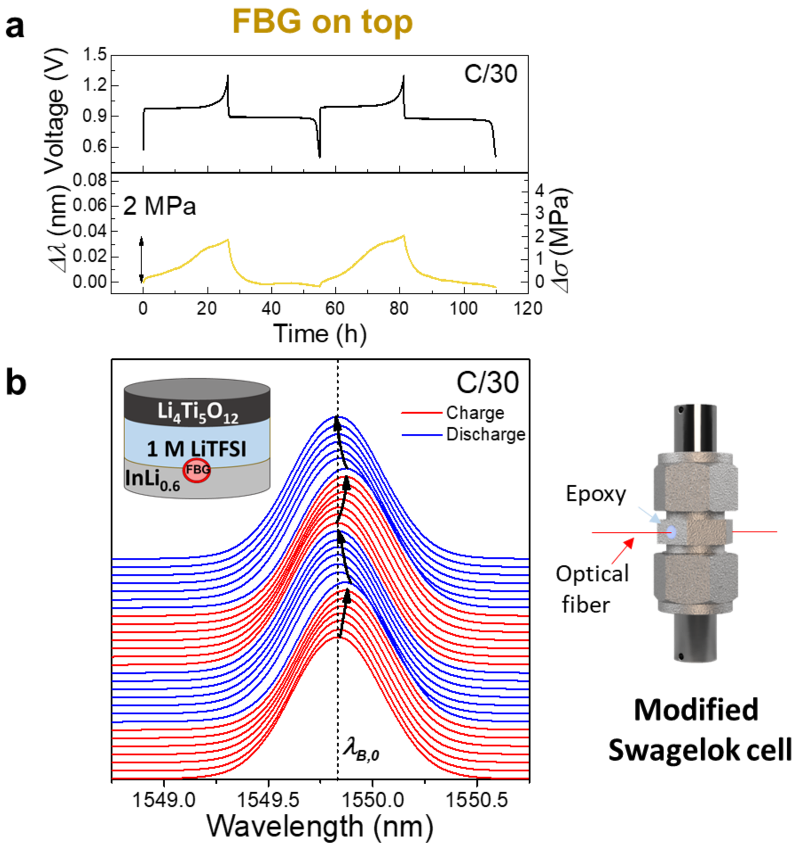

Supplementary Figure 3 | Li-driven stress monitoring in $InLi_x$ || LTO cells with liquid electrolyte via a FBG sensor.
a Time-resolved voltage (top) and $\Delta\lambda$ and $\Delta\sigma$ (bottom) evolution from the FBG sensor of an $InLi_{0.6}$ || LTO cell with liquid electrolyte with the FBG placed at the interface between the anode and the electrolyte contained in the porous separator. The cells were cycled at C/30 (5.83 mA g^{-1}) in a 25 ºC temperature-controlled climatic chamber. The two selected cycles correspond to the 5th and 6th cycle of the cell. **b** The corresponding 2D stack-view of the reflected spectra given by the FBG sensor, for the two cycles selected, are shown in chronological order (from bottom to top). A well-defined Bragg wavelength peak (λ_B) can be tracked and followed upon cycling (indicated by arrows), with the initial Bragg peak denoted as $\lambda_{B,0}$. The scheme of the modified Swagelok cell with the optical fiber implemented is also shown. In comparison to the cell cycled at C/10 (17.5 mA g^{-1}), an increase of ~ 10% in $\Delta\sigma$ is observed (from 1.7 MPa at C/10 to > 2 MPa at C/30) which is consistent with the higher degree of lithiation when moving to a slower C-rate.

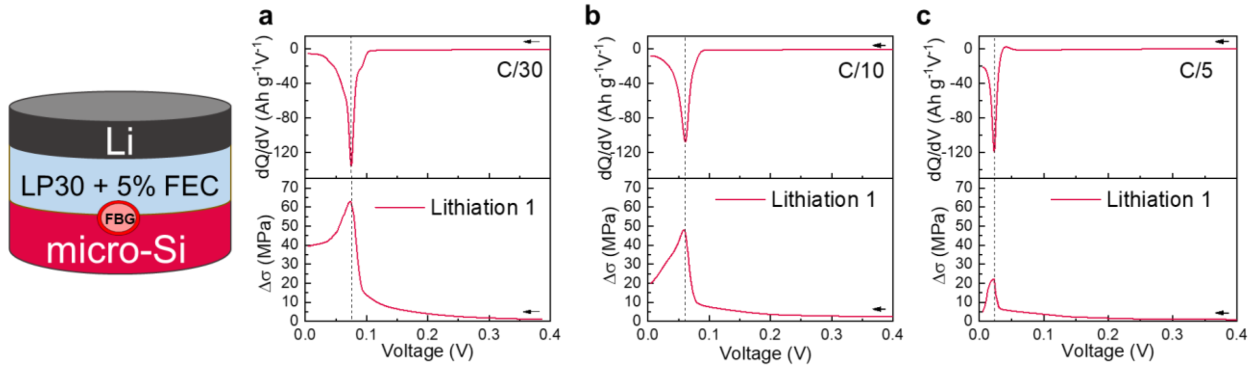

Supplementary Figure 4 | Li-driven stress monitoring via FBG sensor of Li || micro-Si cells with LP30+FEC liquid electrolyte at different C-rates.

Differential capacity vs. potential plot (top) and *Δσ* evolution vs. potential (bottom) for the first silicon lithiation of Li | (LP30+FEC) | micro-Si Swagelok cells with the FBG sensor placed on top of the silicon electrode. The comparison of the first discharge is shown for three diferent C-rates: (**a**) C/30 (120 mA g^{-1}), (**b**) C/10 (360 mA g^{-1}), and (**c**) C/5 (720 mA g^{-1}). Note that the first discharge provides a single peak in the derivative curve, which is correlated to the $\Delta\sigma_{max}$ sensed by the FBG sensor regardless the C-rate.

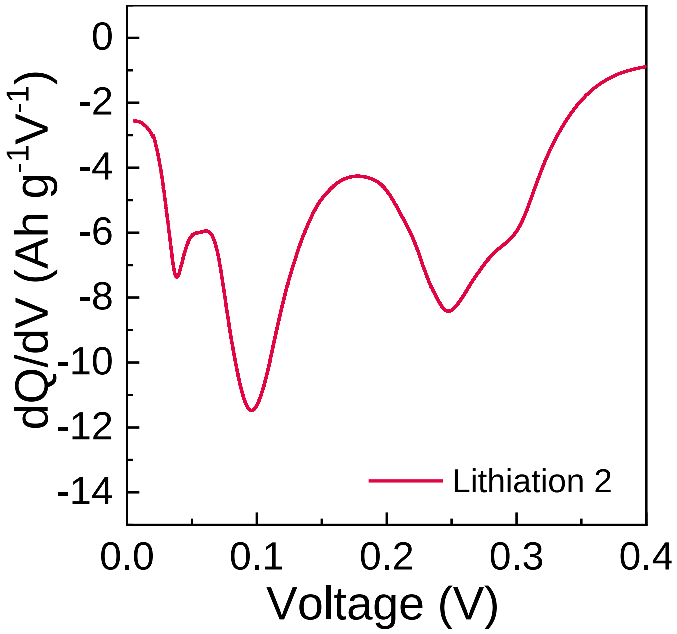

Supplementary Figure 5 | Inset of the second d*Q*/d*V* discharge curve of the micro-Si Swagelok cell.

Differential capacity vs. potential plot for the second discharge of the Li | (LP30+FEC) | micro-Si Swagelok cell with the FBG sensor placed on top of the silicon electrode. Inset of Figure 2b to see in detail the three less intense peaks appearing in comparison to the peak observed for the first lithiation of the silicon. The peak observed at the lowest potential (~ 0.04 V) corresponds to the well documented crystallization of the $Li_{15}Si_4$ phase for electrodes containing micro-Si particles.[1]

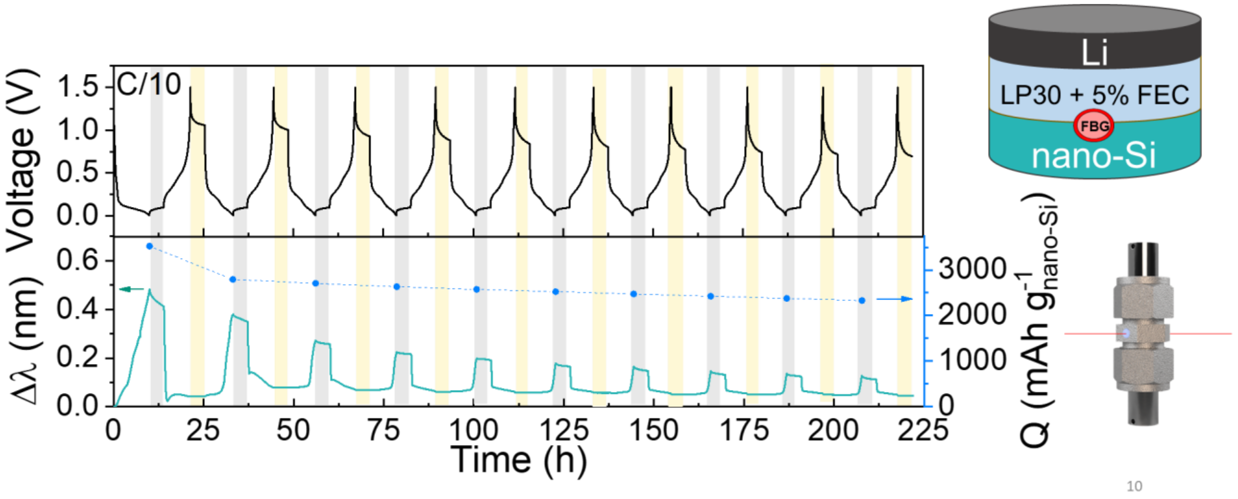

Supplementary Figure 6 | Monitoring Li-driven stress in a Li || nano-Si Swagelok cell with liquid electrolyte. Time-resolved voltage profile (top) and *Δλ* (bottom, left) evolution from the FBG sensor of a Li | (LP30+FEC) | nano-Si Swagelok cell with liquid electrolyte with the FBG placed on the interphase between the silicon electrode/electrolyte. The discharge capacity (bottom, right) is also presented at the end of each discharge for a cell cycled at C/10 (360 mA g^{-1}) in a 25 ºC climatic chamber. As in the case of micro-Si electrodes, the amplitude of the optical signal (*Δλ*) decays with the capacity retention experienced by the cell, which demonstrates their interlinkage.

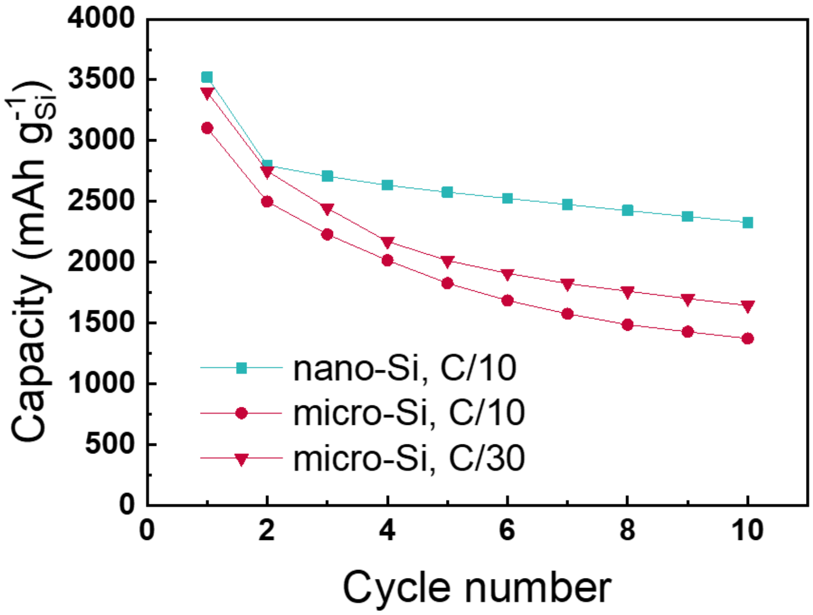

Supplementary Figure 7 | Comparison of the capacity retention for micro-Si and nano-Si based electrodes. Discharge capacity vs. cycle number for the first ten cycles of Li | (LP30+FEC) | Si Swagelok cells with liquid electrolyte containing either nano-Si or micro-Si particles. As reported in the literature, nano-Si based electrodes presents a better capacity retention than the micro-Si ones[2,3]. The cells with nano-Si were cycled at C/10 meanwhile for micro-Si, the capacity retention is presented at two different C-rates: C/10 (360 mA g^{-1}) and C/30 (120 mA g^{-1}). All the cells were cycled in a 25 ºC climatic chamber.

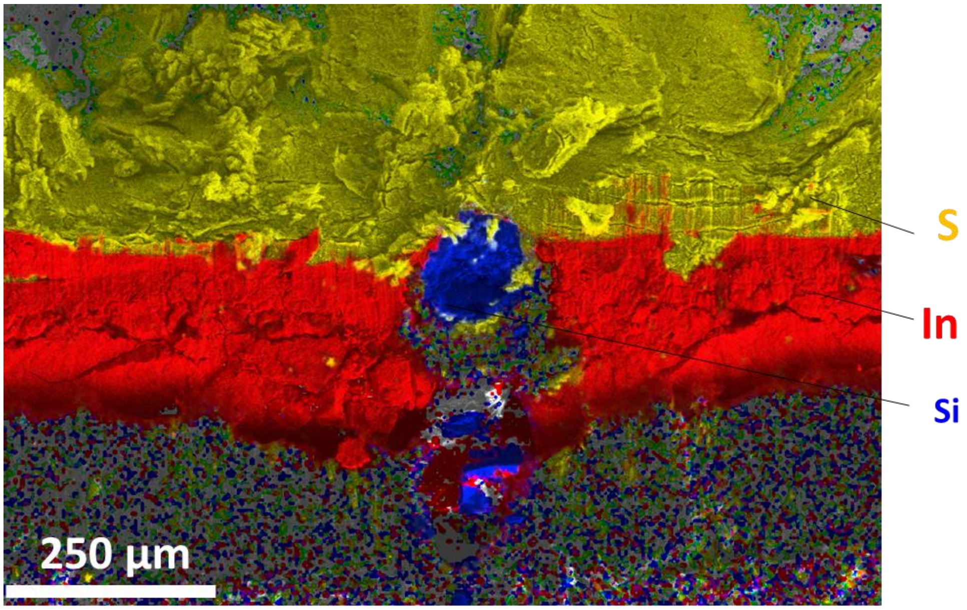

Supplementary Figure 8 | EDX mapping of the cross-section view of the ASSB with the optical fiber.
SEM micrograph together with the corresponding EDX mapping of the cross-section view of a ASSB InLi | Li_3PS_4 | $Li_4Ti_5O_{12}$ battery with the optical fiber embedded in the $InLi_x$ anode. The selected elements to track the different battery components are: S (yellow) for the solid electrolyte, In (red) for the anode and Si (blue) for the silica optical fiber. Note that the optical fiber was placed totally embedded in the anode (see the hole remaining). However, its position changed to the interface between the anode and the solid-state electrolyte during sample preparation, in particular, when sharply cutting the pellet previously embedded in conductive epoxy. The cutting direction with the blade was from the anode to the solid electrolyte. Thus, the optical fiber is suspected to be pulled towards the solid electrolyte.

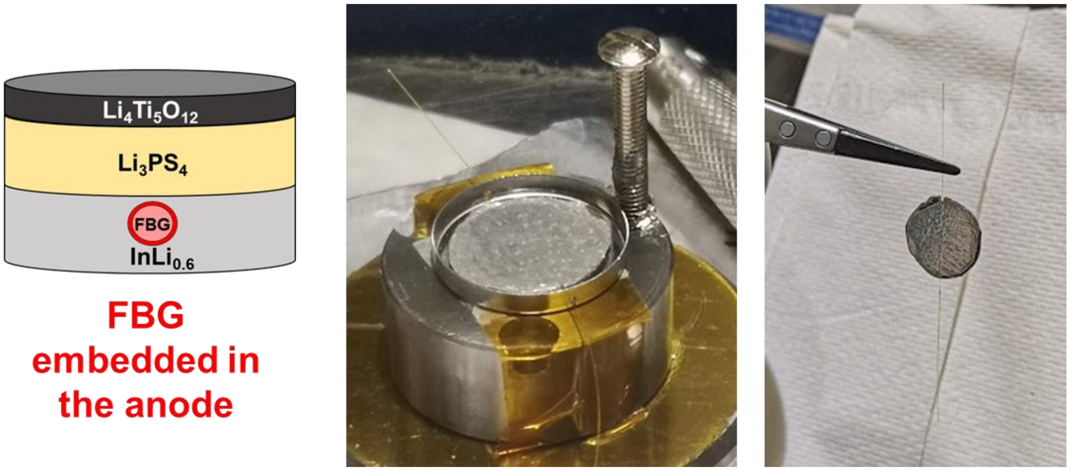

Supplementary Figure 9 | Photographs of a real FBG sensor embedded in the $InLi_x$ anode.
Scheme and real photographs of the optical fiber embedded within the $InLi_x$ anode.

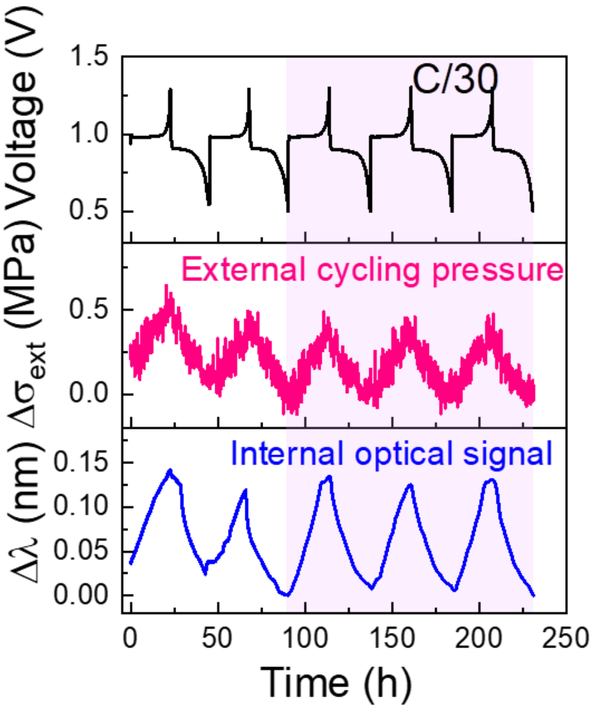

Supplementary Figure 10 | *Operando* Li-driven stress monitoring in $InLi_x$ | LPS | LTO ASSB cell by a FBG embedded in the anode.

Time-resolved voltage (top), external cycling pressure (middle) and internal optical signal (bottom) for the ASSB cycled at C/30 (5.83 mA g^{-1}) and 25 ºC in an *operando* mode. The stabilization of the mechanical and optical signals can be observed after the third cycle due to the mechanical drift/relaxation of the cell. The three cycles shadowed (3rd to 5th cycle) are the ones shown in Figure 4b.

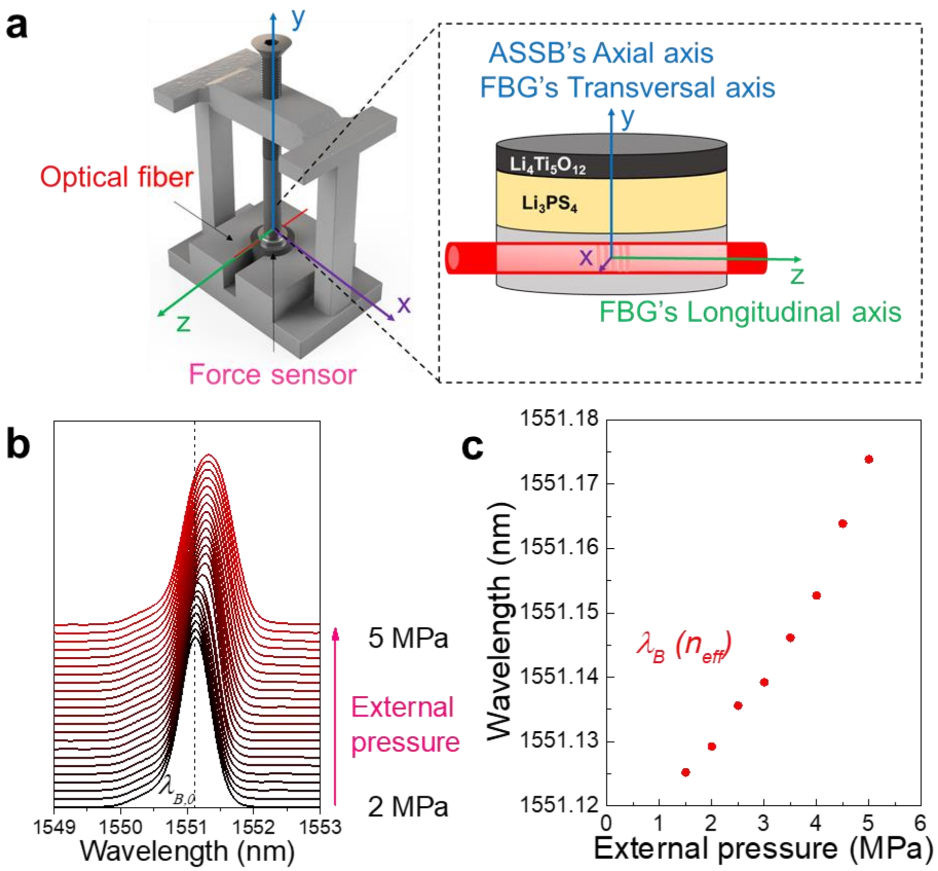

Supplementary Figure 11 | Experimental calibration curve of the ASSB with the FBG embedded in the anode.
a Scheme of the ASSB coin cell with the FBG sensor embedded in the anode. The different axis of the ASSB and the optical fiber are indicated for clarification. **b, c** Experimental calibration curve when the battery is at an open-circuit voltage (OCV). The λ_{max} is recorded while varying the external cycling pressure applied (y-axis in **b**, denoted with an arrow), with the corresponding slope of 0.034 nm MPa^{-1}. The linearity of the sensor is used to obtain the constant rate response of the sensor towards mechanical deformation.

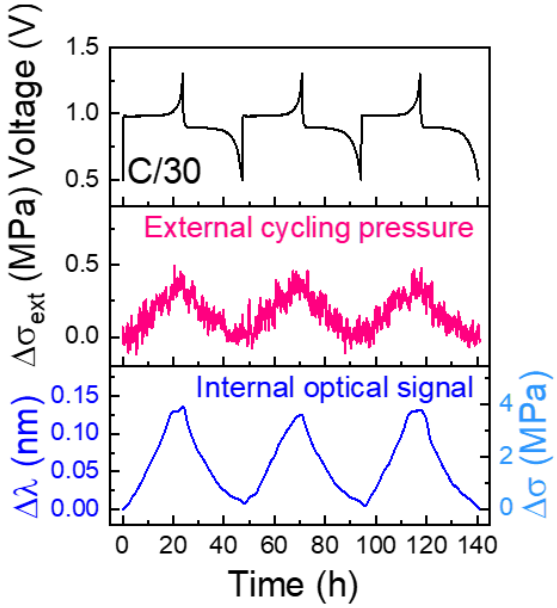

Supplementary Figure 12 | *Operando* Li-driven stress monitoring in $InLi_x$ | LPS | LTO ASSB by a FBG embedded in the anode.

Time-resolved voltage (top), external cycling pressure (middle), internal optical signal ($\Delta\lambda_{max}$, bottom, left) and internal stress evolution by a FBG sensor ($\Delta\sigma$ bottom, right) for the aforementioned ASSB cycled at C/30 (5.83 mA g^{-1}) and 25 ºC in an *operando* mode. The calibration of our FBG sensors by recording the λ_{max} while varying the external cycling pressure applied during battery resting at open-circuit voltage (OCV) is the method selected to show the converted $\Delta\lambda_{max}$ into stress variations ($\Delta\sigma$), and therefore compare the internal with the external stress evolution directly. Following the main text format, the internal stress is noted in the graph by the word "experimental" in Figure 4d.

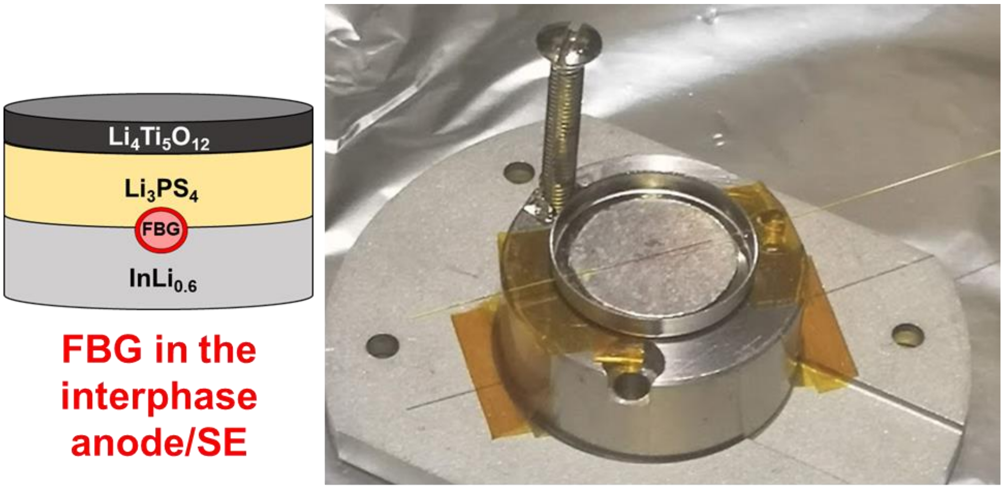

Supplementary Figure 13 | Photographs of a real FBG sensor at the anode/solid electrolyte interface.
Scheme and real photographs of the optical fiber placed at the interface between the $InLi_x$ anode and the solid electrolyte.

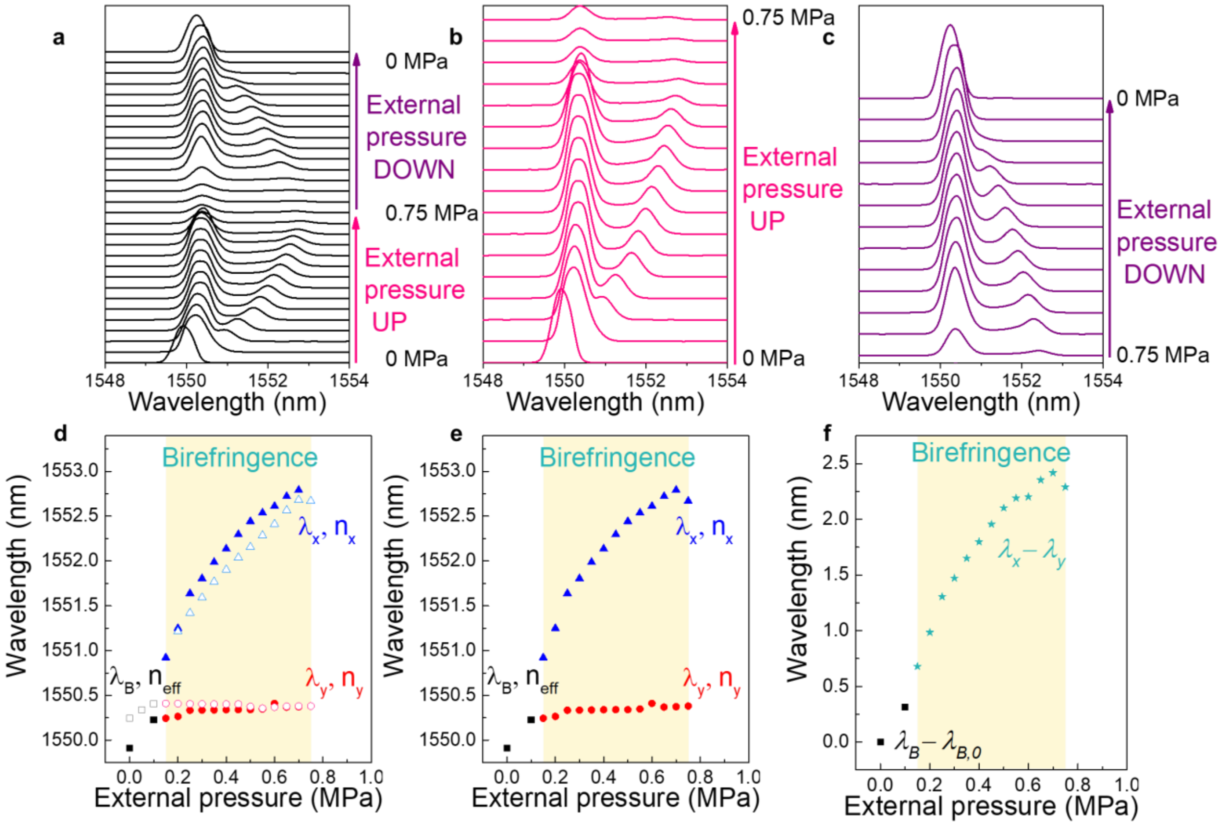

Supplementary Figure 14 | Induced birefringence: FBG sensor between two stainless steel spacers.
a External calibration curve showing the reflected spectra when a transversal load is applied to the FBG sensor. **b, c** show a zoom of the reflected spectra when the applied external pressure is increasing and decreasing, respectively (y-axis direction, denoted with an arrow). The splitting of the Bragg wavelength peak (λ_B) to λ_x and λ_y is known as birefringence. **d, e** and, **f** present the detailed values of λ_B, λ_x, and λ_y vs. the external applied pressure with the force sensor. Before the birefringence region, only one peak (λ_B) is monitored. The birefringence regime is shadowed in light yellow. In (**d**), the filled and hollow scatters show the peak during the pressure increase and decrease, respectively.

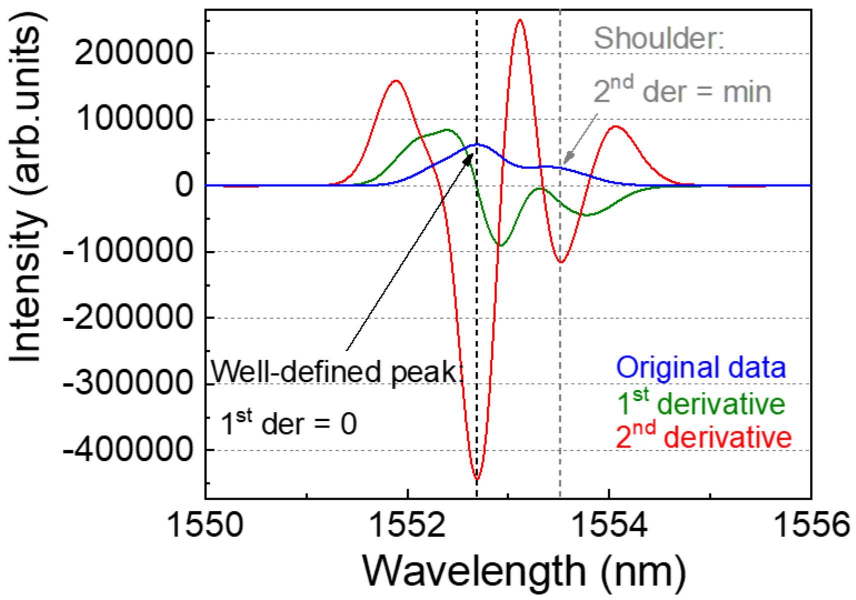

Supplementary Figure 15 | Specific criteria for peak determination.

Criteria to determine a well-defined peak (1st derivative = 0) and a shoulder (2nd derivative = minimum) in the FBG spectra. From our results, we could extract λ_B, λ_x *and* λ_y for the different reflected spectra of the FBG sensor.

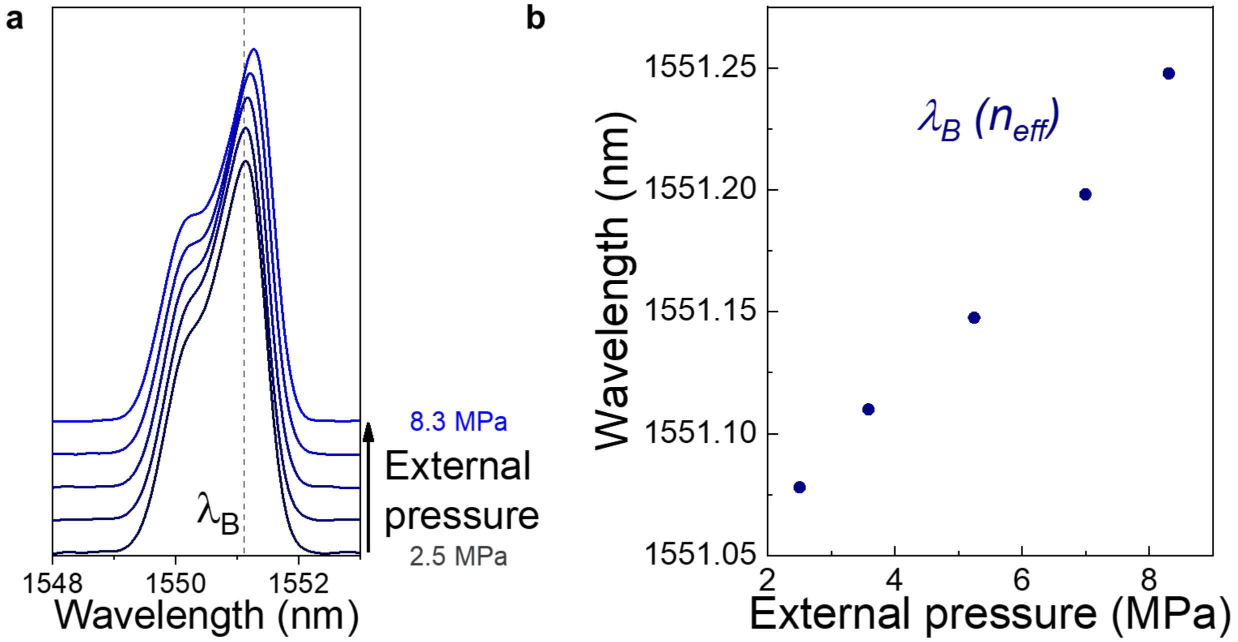

Supplementary Figure 16 | Experimental calibration curve for the symmetrical ASSB, when the external cycling pressure is 2.7 MPa.

a, b show respectively the 2D-stack view of the reflected spectra and the variation of λ_{max} while varying the external cycling pressure applied (y-axis in **a**, denoted with an arrow, and x-axis in **b**) when the battery is at an open-circuit voltage (OCV). The slope of the corresponding linear regression is 0.032 nm MPa^{-1}. The linearity of the sensor is used to obtain the constant rate response of the sensor towards mechanical deformation.

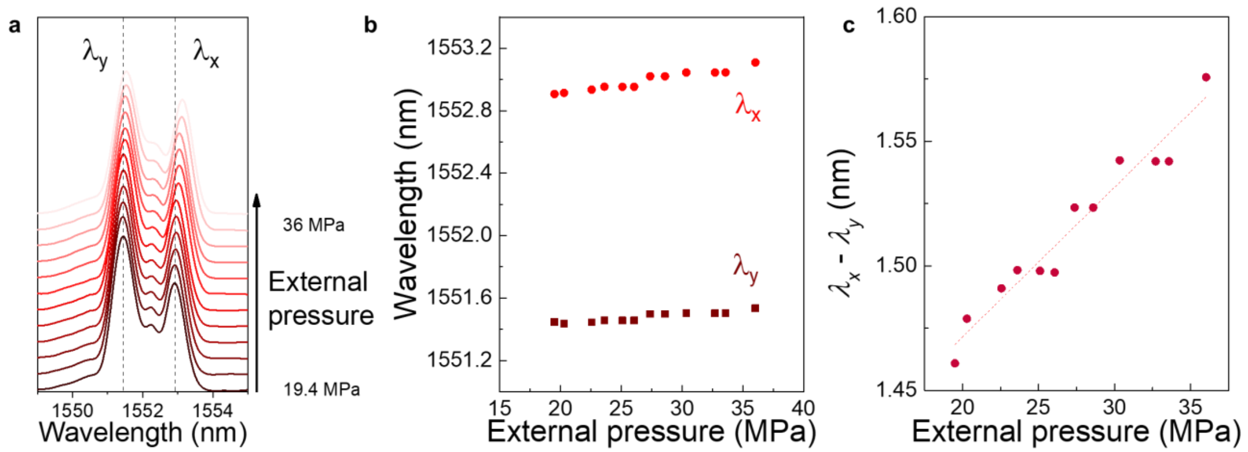

Supplementary Figure 17 | Experimental calibration curve for the symmetrical ASSB, when the external cycling pressure is 21 MPa.

a Experimental calibration curve of the FBG sensor when the ASSB is in a OCV status. The external applied pressure is increased externally (y-axis, denoted with an arrow). **b** Detailed values of λ_x, and λ_y vs. the external applied pressure with the force sensor. The ASSB is in the birefringence regime (λ_x *and* λ_y), with (**c**) the distance between λ_x and λ_y continuously increasing. The slope of the linear fitting in the birefringence regime (19 MPa to 36 MPa) is 0.059 nm MPa^{-1}, and gives directly the information regarding the transversal stress from the FBG sensor, thus, the axial stress of the ASSB (see scheme in Figure 5).

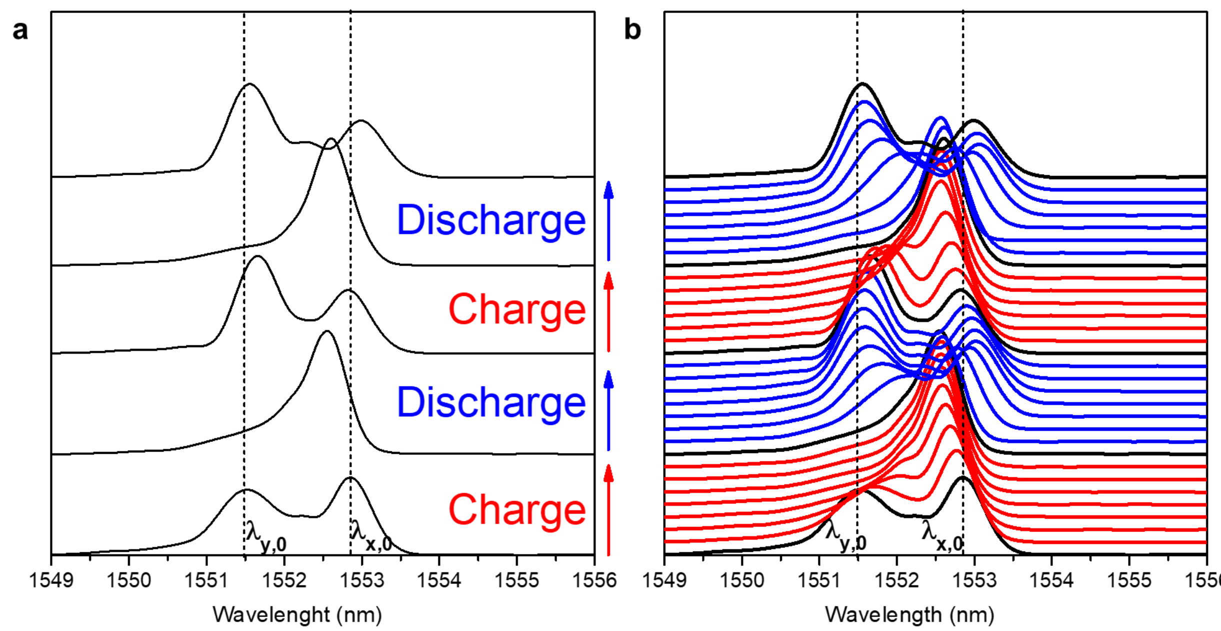

Supplementary Figure 18 | 2D-stack view of the reflected spectra for the symmetrical ASSB with the FBG placed at the cathode/solid-state interface.

a, b present the reflected optical spectra taken in chronological order (y-axis, from bottom to top) obtained during two charge/discharge cycles of the symmetrical ASSB, when the external applied pressure is 27 MPa. The spectra were repeatedly merging in one showing one peak (***λ_B***), and splitting again to λ_x *and* λ_y upon charging and discharging the cell, thus indicating local anisotropic Li-driven stresses that are reversible upon cycling. In this case, the birefringence phenomenon was induced by the electrochemistry.

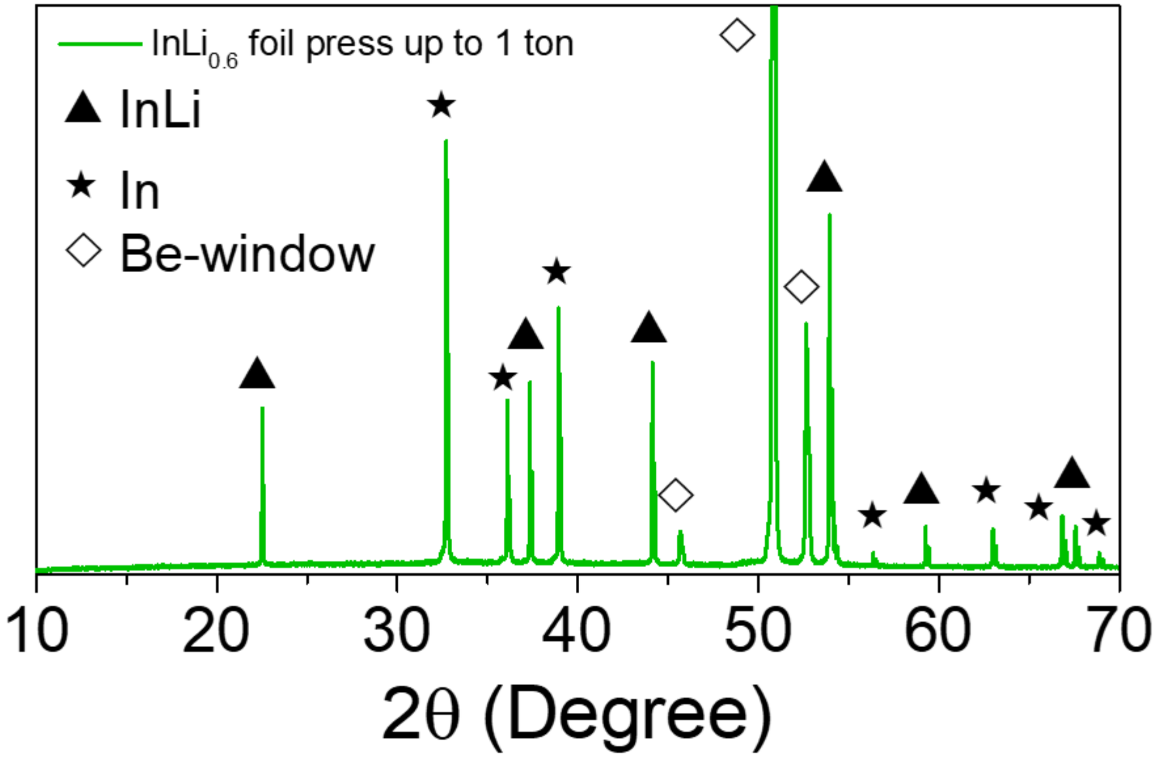

Supplementary Figure 19 | XRD pattern of the Indium-Lithium alloy.
The XRD pattern obtained after pressing one disc of indium foil (99.99 %, Sigma Aldrich, with 0.127 mm thickness) and one of lithium foil (99.9 %, Sigma Aldrich, 0.38 mm thickness) in the size so that the molar ratio between the two metals was approximately InLi0.6 under a hydraulic cold-press up to 1 ton and during 1 minute. The XRD pattern obtained is in accordance with the reference biphasic In-Li alloy, confirming the good formation of the phase. The presence of some indium metal not alloyed is in line with the data previously reported[4].

Supplementary Note 1 | Internal stress estimation from linear strain approximation.
In a very simple approach, this model enables to estimate the maximum stress that a given cell configuration would experience based on the volumetric changes of the electrode materials during operation. This approximation works under two assumptions:

1) Full constraint of the battery within the volume of the testing cell.

2) Mechanical properties of the full battery (represented as the Bulk Modulus K) approximated as the mechanical properties of the SE (LPS in our case, K_{LPS} = 21.4 GPa), given that the SE is the main volumetric component of the stack.

Therefore, the variation of hydrostatic pressure within a fully constrained solid state battery containing LPS as solid electrolyte can be expressed as:

$$|\Delta p| = \varepsilon_{vol} \cdot K_{LPS} \qquad (S1)$$

Which can be further decomposed in three stress components denoted in Equation S1 as σ_{11}, σ_{22} and σ_{33}. Further details are explained in the literature[5,6].

$$|\Delta p| = \frac{1}{3}\left(\sigma_{11} + \sigma_{22} + \sigma_{33}\right) \qquad (S2)$$

Application of the linear strain approximation to the fully constrained $InLi_x$ | LPS | LTO solid state battery from Figure 4:

LTO | LPS pellet dimensions
Thickness = 0,60 cm
Area = 1,33 cm^2

Anode volume
Anode composition: 6,53 mg Li (0,94 mmol) + 176,5 mg In (1,55 mmol). When the two metals are pressed together, the following alloying reaction takes place:

In ($\tilde{V}_{In}$= 15,7 cm^3 mol^{-1}) + Li^+ + e^- → InLi ($\tilde{V}_{LiIn}$= 23,6 cm^3 mol^{-1}) $\qquad Reaction\ S1$

After alloying reaction, the anode composition is the following: 0,94 mmol InLi + 0,61 mmol In
Taking into account the molar volumes of reagent and product in Reaction S1, the total anode volume results:

$V_{A\text{-}Tot}$ = 0,0316 cm^3 of In/InLi

The anode was evenly distributed onto the current collector (1,76 cm^2 ,15 mm diameter circle) but the LTO | LPS pellet surface was 1,33 cm^2 (13 mm diameter circle), therefore, the effective anode volume can be calculated as:
(1,33/1,76) x 0,0316 cm^3
$V_{A\text{-}eff}$ = 0,0240 cm^3

Battery dimensions
The volume of the battery assuming total constrain can be calculated as volume of LTO | LPS pellet + effective anode volume
= (0,6 cm * 1,33 cm^2)+ 0,0240 cm^3
V_{cell} = 0,822 cm^3

Volume change upon cycling
Cell capacity: 130 mAh g^{-1} → 20 mg cathode composite (6 mg LTO) = 0,779 mAh of active material = 2,8 C = 3*10^{-5} mol Li^+ = moles of In converted in InLi according to the hemi-reaction; (Reaction S1)
From the molar volumes in Reaction S1 the maximum volume variation of the stack can be calculated as:
ΔV_{cell} = 0,000237 cm^3

Volumetric Strain
ε_{vol} = ΔV_{cell} / V_{cell}
ε_{vol} = = 0.000237 / 0.822

Going back to Equation S1, for this system:

$$|\Delta p| = \varepsilon_{vol} \cdot K = \left(\frac{0.000237}{0.822}\right) \cdot 21.4 \cdot 1000 = 6\ MPa$$

Therefore, the hydrostatic pressure within the battery can be estimated as $|\Delta p|$= 6 MPa and the axial component of the stress as σ_{11}= 6 MPa.

SUPPLEMENTARY REFERENCES

1. Obrovac, M. N. & Krause, L. J. Reversible Cycling of Crystalline Silicon Powder. *J. Electrochem. Soc.* **154**, A103 (2007).
2. Graetz, J., Ahn, C. C., Yazami, R. & Fultz, B. Highly Reversible Lithium Storage in Nanostructured Silicon. *Electrochem. Solid-State Lett.* **6**, A194 (2003).
3. Li, H. A High Capacity Nano-Si Composite Anode Material for Lithium Rechargeable Batteries. *Electrochem. Solid-State Lett.* **2**, 547 (1999).
4. Santhosha, A. L., Medenbach, L., Buchheim, J. R. & Adelhelm, P. The Indium−Lithium Electrode in Solid-State Lithium-Ion Batteries: Phase Formation, Redox Potentials, and Interface Stability. *Batteries & Supercaps* **2**, 524–529 (2019).
5. Zhang, W. *et al.* (Electro)chemical expansion during cycling: monitoring the pressure changes in operating solid-state lithium batteries. *J. Mater. Chem. A* **5**, 9929–9936 (2017).
6. Koerver, R. *et al.* Chemo-mechanical expansion of lithium electrode materials – on the route to mechanically optimized all-solid-state batteries. *Energy Environ. Sci.* **11**, 2142–2158 (2018).
